# Recent Progress in Terrestrial Biota Derived Antibacterial Agents for Medical Applications

**DOI:** 10.3390/molecules29204889

**Published:** 2024-10-15

**Authors:** Todorka G. Vladkova, Younes Smani, Boris L. Martinov, Dilyana N. Gospodinova

**Affiliations:** 1 Department of Polymer Engineering, University of Chemical Technology and Metallurgy, 8 “Kl. Ohridski” Blvd, 1756 Sofia, Bulgaria; 2 Andalusian Center of Developmental Biology, CSIC, Junta de Andalusia, University of Pablo de Olavide, 41013 Seville, Spain; ysma@upo.es; 3 Department of Molecular Biology and Biochemical Engineering, Andalusian Center of Developmental Biology, CSIC, Junta de Andalusia, University of Pablo de Olavide, 41013 Seville, Spain; 4 Department of Biotechnology, University of Chemical Technology and Metallurgy, 8 “Kl. Ohridski” Blvd, 1756 Sofia, Bulgaria; brsmartinov@uctm.edu; 5 Faculty of Electrical Engineering, Technical University of Sofia, 8 “Kl. Ohridski” Blvd, 1756 Sofia, Bulgaria; dilianang@tu-sofia.bg

**Keywords:** terrestrial biota, antibacterial products, antibacterial compounds, bacteriocines, biosynthesized nanoparticles, combination treatments

## Abstract

Conventional antibiotic and multidrug treatments are becoming less and less effective and the discovery of new effective and safe antibacterial agents is becoming a global priority. Returning to a natural antibacterial product is a relatively new current trend. Terrestrial biota is a rich source of biologically active substances whose antibacterial potential has not been fully utilized. The aim of this review is to present the current state-of-the-art terrestrial biota-derived antibacterial agents inspired by natural treatments. It summarizes the most important sources and newly identified or modified antibacterial agents and treatments from the last five years. It focuses on the significance of plant- animal- and bacteria-derived biologically active agents as powerful alternatives to antibiotics, as well as the advantages of utilizing natural antibacterial molecules alone or in combination with antibiotics. The main conclusion is that terrestrial biota-derived antibacterial products and substances open a variety of new ways for modern improved therapeutic strategies. New terrestrial sources of known antibacterial agents and new antibacterial agents from terrestrial biota were discovered during the last 5 years, which are under investigation together with some long-ago known but now experiencing their renaissance for the development of new medical treatments. The use of natural antibacterial peptides as well as combinational therapy by commercial antibiotics and natural products is outlined as the most promising method for treating bacterial infections. In vivo testing and clinical trials are necessary to reach clinical application.

## 1. Introduction

Increasing resistance to traditional antibiotics is expanding across the world at an alarming rate. Expecting to be the next global pandemic, antimicrobial resistance (AMR) is already recognized as one of the most serious threats to human health [1,2].

Conventional antibiotic and multidrug treatments have become less and less effective. Every year, various microbial pathogens cause infectious diseases for many humans with lethal issues for some of them. It was estimated that bacterial AMR was directly responsible for 1.27 million and contributed to 4.95 million global deaths in 2019 [2]. The first World Health Organization (WHO) regional assessment on health predicts that 5.2 million people in the Western Pacific Region will die between 2023 and the end of 2030 because of drug-resistant bacterial infections [3]. Without new and better treatments, the number of global deaths is expected to rise to 10 million by 2050, which is higher than deaths attribute to cancer (8.2 million) or diabetes (1.5 million) [4,5,6,7]. In addition to death and disability, AMR has significant economic costs. The World Bank estimates that AMR could result in losses to gross domestic product from 1 trillion to 3.4 trillion USD per year by 2030, with additional healthcare costs of 1 trillion USD by 2050 [8]. According to a 2021 WHO report, the pool of new antimicrobial compounds in clinical testing is limited. Only six of thirty-two antibiotic agents in the clinical establishment (that meet the WHO listing of critical pathogens) were categorized as novel in the year 2019 [9]. During the period 2019–2023, FDA approved, according to our checking, the following antibacterial drugs: Likmez, Voquezna, Twyneo, Xaciato, Zilxi, Recarbrio, and Fetroja.

In early 2017, the WHO published the Priority Pathogens List of bacteria that are most frequently involved in multidrug-resistant infections. The updated 2024 WHO Bacterial Priority Pathogens list (WHO BPPL) covers 24 pathogens, spanning 15 families, which require the greatest action with supplementary antibacterial therapies. Among them are Gram-negative bacteria resistant to last-resort antibiotics, multidrug-resistant mycobacterium tuberculosis, and other high-burden resistant pathogens such as *Salmonella*, *Shigella*, *Neisseria gonorrhoeae*, *Pseudomonas aeruginosa*, and *Staphylococcus aureus* [10].

Global concern for human morbidity and mortality caused by multidrug-resistant organisms require urgent development of creative and innovative approaches, from chemical identification and analysis to an assessment of bioactivity of new antimicrobial agents [11,12,13].

Many investigations are devoted to understanding the mechanism of different bacterial species drug resistance and thus to help the development of new approaches mitigating the problems of AMR- [1,14,15,16,17,18,19]. Mir et al. [20] and, lately, Helmy et al. [21] discuss alternative antibiotic approaches that might be used to control AMR, including probiotics, prebiotics, antimicrobial peptides, small molecules, organic acids, essential oils, bacteriophages, fecal transplants, and nanoparticles. With the ability to kill Gram-negative and Gram-positive bacteria or to inhibit their growth, numerous antibacterial peptides (ABPs) have already demonstrated potential as novel therapeutic agents. However, as drugs, they have some undesirable properties, including instability and toxicity, that should be overcome for their clinical translation [22].

The lack of good-quality antibacterial drugs remains a major problem, and the discovery of effective and safe antibacterial agents has already become a global priority. Natural biologically active products have played a vital role in human survival for millennia. Returning to them is a relatively new current trend. The interest in natural biologically active products has increased because of their high efficiency, structural diversity, potential to offer lead compounds, low toxicity, and lack or delayed development of resistance. Antibacterial activity toward Gram-negative and Gram-positive bacteria for many natural compounds isolated from plants, animals and microorganisms was experimentally proved [23], but clinical developments are insufficient [24].

Natural anti-biofilm agents from marine [25,26] and terrestrial biota [27], as well as antimicrobial agents from marine biota, including antioxidant active substances [28,29,30], were a subject of our previous studies.

The aim of this review is to present the current state-of-the-art of terrestrial biota-derived antibacterial agents inspired by natural treatments. It is a summary of the most important sources of newly identified or modified antibacterial agents and treatments over the last five years to the present. The focus of this review is the significance of herbs and plant-derived, animal-derived, and bacteria-derived biologically active agents as powerful alternatives to antibiotics, as well as the advantages of utilizing natural antibacterial molecules alone or in combination with antibiotics. It includes natural antibacterial agents derived from plants, animals and bacteria, their experimentally found or machine (in silico) predicted antibacterial activity, as well as their potential advantages, limitations, and disadvantages, as well as any modifications to reduce the latter. The expectation of this review is to aid the future development of more effective antibacterial drugs and approaches to combating the rising number of bacterial infections.

## 2. Approaches to Avoid/Mitigate the Bacterial Resistance

The medical applications of terrestrial biota derived antibacterial agents are associated with treatment of antibiotic-resistant bacterial infections. Therefore, potential places for natural products, currently used or under study, as an approach to overcome/mitigate antibacterial resistance are shortly presented here [11].

**Figure 1:**
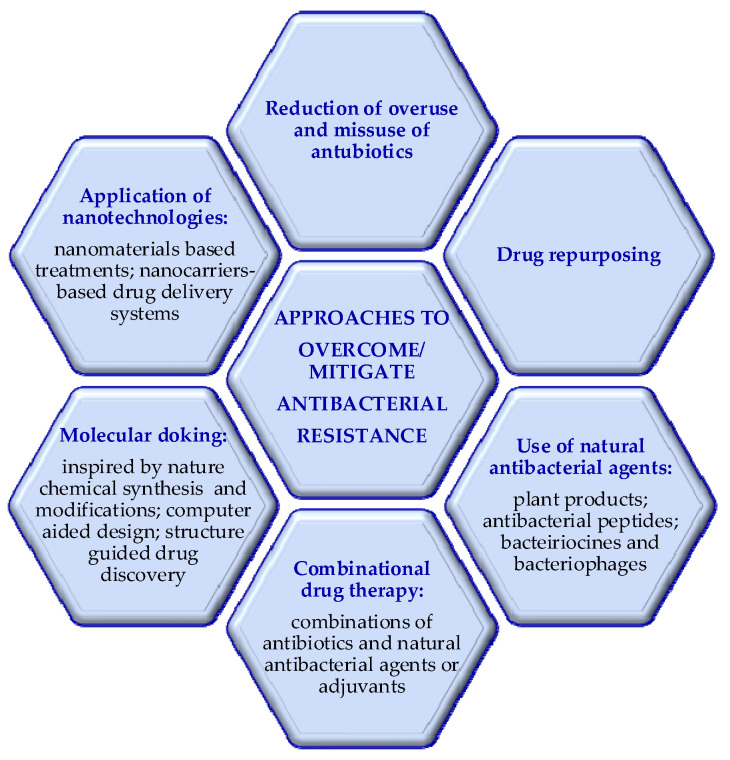
Approaches to overcome bacterial resistance: reduction overuse and misuse of antibiotics; drug repurposing; use of natural antibacterial agents; combinational drug therapy; molecular docking and application of nanotechnologies.

As evident in Figure 1, antibacterial approaches include the following: reducing overuse/misuse of antibiotics [11]; drug repurposing [31]; treatments by nanomaterials and the use of nano-delivery systems [32]; application of natural antibacterial agents, bacteriophage therapy, immunomodulation, etc. [33]; molecular docking (ethno-pharmacological approaches; synthetic strategies inspired by nature; chemical modifications of existing natural antimicrobials, semi-synthesis; computer-aided design, etc.) [11,34]; combination therapy (combinations of phytochemicals and antibiotics; antibiotics and adjuvants) [35,36,37,38,39,40].

### 2.1. Use of Natural Antibacterial Agents: Plant Products, Antibacterial Peptides, Bacteriocines and Bacteriophages

The study of natural products as alternatives or adjuvants to current antibiotics becomes a more and more popular approach in medical treatments due to their generally low toxicity and chemical diversity, which provides important therapeutic effects and make the microbes unable to copy themselves for creating resistance [23]. Bacteriophage therapies and peptide therapies are studied as novel options to control the development of multidrug resistance (MDR). CRISPR, an innovative genome editing technology, offers multiple safeguard applications to overcome the different challenges of MDR. Immunotherapy is a way to improve the host defense and combating issues of bacterial drug resistance using mainly plant products [11].

### 2.2. Combination Drug Therapy

This approach includes the combination of antibiotics and phytochemicals or antibiotics and adjuvants, among others. Herbal extracts, essential oils and isolated pure compounds are reported to act synergistically with existing antibiotics and to increase their activity [35]. Theoretical and practical framework are outlined for the development of effective combinations of antibiotics and antibiotics with non-antibiotic compounds [36,37]. Combination therapies of existing antibiotics and adjuvants are accepted as promising for mitigating the problem of AMR [38,39].

The combination of different drugs offers many advantages over their use as individual chemical moieties, namely: reduction of dosage; fewer side effects; reduced risk for the development of drug resistance; better combined response; wide-spectrum antibacterial action; and ability to attack simultaneously multiple target sites. An appropriate combination approach provides a pathway to the development of antimicrobial therapeutics with broad-spectrum antibacterial action, bactericidal instead of bacteriostatic mechanisms of action, and better efficacy against multidrug-resistant bacteria [40].

### 2.3. Nanotechnologies

Nanotechnologies are an emerging, widely popular approach for the treatment of multidrug-resistant infectious diseases. The use of nanomaterials, mainly biologically synthetized metal, metal oxide, and composites nanoparticles, as well as based on nanocarriers as a drug delivery system is a relatively new trend for the following: to improve pharmacokinetics, stability, and solubility; to reduce toxicity; and to provide controlled release of therapeutic agents or other compounds at the target site. Some nanoparticles extracted from natural products, such as *Glycyrrhiza glabra* L. green tea, *Allium sativum* L. and *Ginkgo biloba* L., demonstrate good antimicrobial potential [41]. Liposome-based nanoparticles can restore the potency of antibiotics such as ceftazidime, imipenem, and cefepime against multidrug-resistant *P. aeruginosa*, amikacin for *K. pneumoniae*, and chloramphenicol for MRSA-65 by effective drug administration [32,41,42].

### 2.4. Molecular Docking Inspired by Nature

Inspired by natural chemical synthesis, chemical modifications of existing natural antimicrobials, semi-synthesis, and computer-aided design are relatively new approaches to the development of novel antibacterial agents. The antimicrobial characteristics of some naturally occurring chemicals produced by bacteria, plants, and animals are studied for use as lead molecules [43]. Structure-guided drug discovery [44] and ethno-pharmacological approaches also contribute to molecular docking. Their goals are to update knowledge on natural antimicrobial products and their ethno-medicinal uses in preventing and treating infections, as well as future research directions for the discovery of new antibiotics from natural products [45]. For example, developed in 1930s, the efficacy of maggot debridement therapy (involving the use of maggots of the green-bottle fly) on wounds to remove necrotic, sloughy and/or infected tissue, is now studied on *S. aureus* and *P. aeruginosa* in diabetic foot ulcers [46]. Ethnobotany and plant natural products are currently discussed as a promising source of antibacterial lead compounds that could help with new drug discovery [47].

The demand of new antibiotics, such as antimicrobial peptides, nanoparticles, combinatorial therapies, and structure-guided drugs, is emerging as the most important future strategy in the design of antibacterial agents, including agents that can selectively interact with a target site (a gene or a cellular process) or a specific pathogen [40]. Natural antimicrobial agents acting on different mechanisms are now under development to attack the development of the bacteria immune defense elements and mechanisms, such as virulence factors [48], immune system elements [49,50,51], to directly inhibit bacteria’s immune system [52], or targeting oxidative stress in bacteria [53].

## 3. Terrestrial Sources of Antibacterial Agents

**Figure 2:**
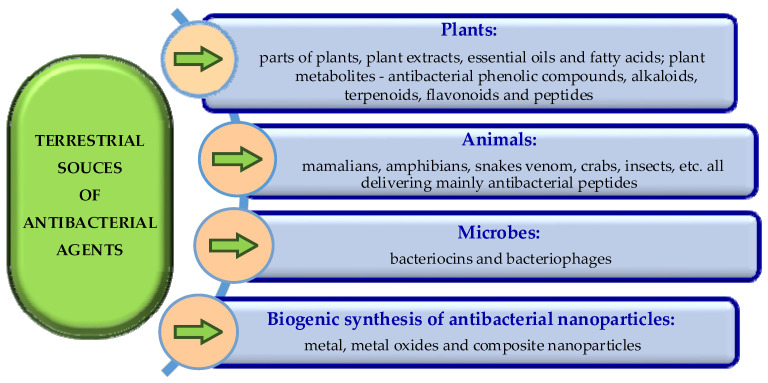
Terrestrial sources of antibacterial agents: plants, animals, microbes, and biogenic synthesis of antibacterial nanoparticles.

Terrestrial sources of antibacterial agents are plants, animals and even bacteria (Figure 2). The most promising natural compounds used to combat bacteria include plant extracts, essential oils, small antimicrobial peptides of animal origin, bacteriocins, and various groups of plant compounds (triterpenoids, alkaloids, phenols, and flavonoids) with antimicrobial and antiviral activity [23].

Antioxidants such as polyphenols, vitamins, and carotenoids, derived from natural sources and dominantly involved in boosting the defense system of organisms are accepted as natural antibiotics [54]. Inspired by nature, solutions are promising for the discovery of new antibiotics and antibacterial treatments [55]. Currently, natural products continue to be among the most essential resources of modern therapeutics, since they have a wide range of chemical and functional variability, as well as fewer side effects, synergy, and the capacity to combat drug tolerance. Secondary metabolites synthesized by plants, animals, and microbes can be used to isolate biologically active molecules [56].

Natural products provide a significant number of new chemical compounds that exhibit biological or pharmacological properties with therapeutic characteristics (lead compounds) and integral components of currently accessible drug formulations. The two crucial elements that propel natural materials from precursors to medicines are pharmacological activity and drug ability. Therefore, structural traits are under investigation for their adjustment to a corresponding application [1]. Both short-term and long-term solutions are proposed to overcome the limitations in various research sectors with the aim to bridge the gap between academic, industrial, and political stakeholders, and to unite interdisciplinary expertise to benefit from the development of future generations of antibiotics [57].

The antibacterial potential of natural products isolated from plants, animals, and bacteria, either alone or in combination with conventional antibiotics against multidrug-resistant pathogens is of especial interest. Prebiotics, probiotics, synbiotics, bacteriophages, nanoparticles, and bacteriocins are now presented as new tools supporting the progress of effective antibiotics to combat antibiotic-resistant bacteria [58]. The development of bacterial vaccines is also of current interest [59].

As is evident from Figure 2, plant-originating antibacterial agents include plant extracts, essential oils and fatty acids, as well as antibacterial compounds, such as phenols, alkaloids, terpenoids, flavonoids, and peptides. Animal-originating antimicrobial agents are mainly peptides. Bacteriocines and bacteriophages originate from bacteria. Biogenic synthesis is a relatively new source of antibacterial nanoparticles. All these antibacterial agents and their sources are presented in more detail in the next sections.

### 3.1. Plant-Derived Antibacterial Products

Since ancient times, the world has used plants as medicine. Herbs, herbal components, and materials containing several parts of plants or other plant-based compounds were traditionally used to treat multiple health ailments. Medicinal plants, including garlic (*Allium sativum*), ginger (*Zingiber officinale*), green tea (*Camellia sinensis*), St. John’s wort (*Hypericum perforatum*), black cumin (*Nigella sativa*), licorice (*Glycyrrhiza glabra*), Mongolian milkvetch (*Astragalus membranaceus*), and purple coneflower (*Echinacea* spp.), possess a notable history of efficacy in managing microbial diseases. Exhibiting noticeable immune-boosting properties and potential to combat bacterial pathogens, these plants have been thoroughly searched and effectively utilized [60].

The use of herbal antibacterial (clove, portulaca, tribulus, eryngium, cinnamon, turmeric, ginger, thyme, pennyroyal, mint, fennel, chamomile, burdock, eucalyptus, primrose, lemon balm, mallow, and garlic), extracts or derived active components, instead of synthetic chemical drugs is increasing because of their fewer side effects, strong antimicrobial properties and primary healthcare benefits [61]. Five herbal antimicrobials, echinacea, manuka, thyme, olive leaf, and astragalus, have been reported to fight infections naturally, [62]. Echinacea, also known as coneflower, is called the “toothache plant” in many Native American cultures, because of its antibiotic and antimicrobial action. Experts for treating toothaches [61], recommend infusing a few drops of fresh leaves of this herb to create a mouthwash concoction. Ginger, clove, garlic and turmeric can also be used in different ways to reduce tooth infections [63].

Strain-specific activity was found for curcumin (from *Curcuma longa* L.), that was for a long time known for its antibacterial properties. Experimental results confirm a much greater sensitivity of Gram-positive than Gram-negative bacteria and suggest that numerous clinical strains of widespread pathogens have poor sensitivity to curcumin (the MICs of the multidrug-resistant types of *Staphylococcus aureus*, *S. haemolyticus*, *Escherichia coli*, and *Proteus mirabilis* are high, ≥2000 µg/mL). However, curcumin is effective against some species and strains: *Streptococcus pyogenes* (median MIC = 31.25 µg/mL), methicillin-sensitive *S. aureus* (250 µg/mL), *Acinetobacter lwoffii* (250 µg/mL), and individual strains of *Enterococcus faecalis* and *Pseudomonas aeruginosa* (62.5 µg/mL). Based on these results, curcumin is classified as a promising antibacterial agent with a very selective activity [64]. Curcumin blocks bacterial growth owing to its structural characteristics and the generation of antioxidant products. It can inhibit bacterial virulence factors and bacterial biofilm formation and can prevent bacterial adhesion to host receptors through the bacterial quorum sensing regulation system. As a photosensitizer, curcumin acts under blue light irradiation to induce photo toxicity and inhibit bacterial growth. Moreover, it can exert a synergistic antibacterial effect with other antibacterial substances [65]. In silico design and mechanistic study of niosomes (non-ionic surfactant-based vesicles) encapsulated curcumin suggests that it is active against multidrug-resistant *Staphylococcus aureus* biofilms and can be extended to be active against biofilms of other pathogens [66].

Simultaneously with direct antibacterial activities, some phytoproducts demonstrate in vitro synergistic effects in combination with conventional antibiotics [67]. It is generally agreed that medicinal plants could contribute to avoiding a crisis with microbial resistance to conventional antibiotics and multidrug treatments because of their attractive features. Plants are readily available and cheap, extracts or compounds from plants often demonstrate high activity against bacterial pathogens and they rarely have severe side effects. The large variety of plant-derived compounds provides diverse chemical structures that may supply both novel mechanisms of antimicrobial action and new targets within the bacterial cell. The rapid development of modern biotechnologies opens ways for obtaining bioactive compounds in environmentally friendly and low-toxic conditions [68]. The rich and unique chemo diversity, the worldwide distribution and ease of access, the various antibacterial modes of action, and the proved clinical effectiveness of plant extracts are a major strength of natural plant products as a promising source of antibacterial lead compounds [46].

Plant-derived antibacterial products, such as extracts, oils, fatty acids, biosurfactants, and a large variety of biologically active compounds, are under intense study for their potential to fight AMR as adjuvants to antibiotics or as lead molecules for the development of new, more efficient antibacterial drugs.

#### 3.1.1. Antibacterial Plant Extracts

**Figure 3:**
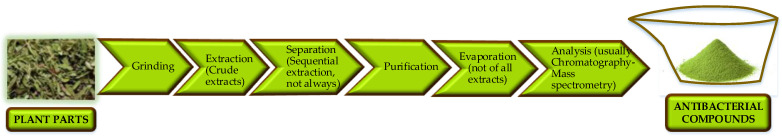
Principle sketch of plant extracts preparation, including grinding of plant parts, extraction, separation, purification, evaporation, and analysis of the extracted antibacterial materials.

Antibacterial plant extracts are prepared via technologies whose simple sketch is presented in Figure 3. The extraction technologies usually include several steps, starting with the grinding of plant parts, followed by crude extract preparation, separation by sequential extraction, purification, evaporation, analysis, and collection of the obtained antibacterial material. In some cases, grinding is to a very fine powder that can be directly applied as antibacterial material. In most cases, the grinded powder passes crude extraction using different solvents/solvent systems. Together with the type of extraction technology and its parameters, the solvents are very important for the total yield and composition of the extract. Some crude extracts demonstrate high antibacterial activity and, after purification, drying and analysis, they are used as antibacterial agents. Drying can be avoided if the crude extract will be used in liquid form.

The crude extracts contain a mixture of natural biologically active compounds and therefore they usually pass sequential extraction to isolate different components of the mixture. Sequential extracts are further processed similarly to the crude extracts; they pass purification, evaporation (if it is necessary), and analysis to obtain the final product [69].

Plant extracts often demonstrate high activity against bacterial pathogens. The screening of the antimicrobial potential of four different plant extracts against twelve pathogenic microorganisms and two reference bacterial strains demonstrates that most extracts exhibit different antimicrobial activity in vitro. The extracts of *Oxalis corniculata* are most efficient against Gram-negative bacteria (*Escherichia coli*, *Salmonella Typhi*, *Klebsiella pneumoniae*, and *Citrobacter koseri*). The methanol extracts of *Artemisia vulgaris*, *Cinnamomum tamala*, and *Ageratina adenophora* show efficacy against *Staphylococcus aureus* [70].

In vitro and in vivo evaluation of the anti-salmonella effect of pectin extracts and hydrolysates from “*Cas mango*” (*Spondias dulcis*) showed inhibition zones from 12.0 to 15.0 mm (for a pectin solution of 100 μg/ml, by disk diffusion test) and MIC values for the different strains of *Salmonella* spp. from 5.68 to 44.45 μg/mL. Treatment with these extracts of mice infected with *Salmonella* spp. prolongs their lifespan [71].

In vitro tests have demonstrated the inhibition of *Staphylococcal* pathogenesis by witch hazel and green tea extracts [72], the antimicrobial activity of *Hibiscus* acid and chromatographic fractions from *Hibiscus Sabdariffa Calyces* against multidrug-resistant pathogenic bacteria [73], as well as the antibacterial potential of ethanol extracts of neem leaves (*Azadirachta indica*) against uropathogens producing beta-lactamase enzymes [74].

The influence of the extraction solvent on the phenolic profile and bioactivity of two *Achillea species* was shown. The ethanol extract of *A. abrotanoides Vis*. (yarrow) demonstrated more significant antimicrobial activity against Gram-positive bacteria *Enterococcus faecalis* than the antibiotic ampicillin. The very high activity against *E. faecalis* was ascribed to the estimated high concentration of the flavanone, naringenin, in the ethanolic extract [75].

The antimicrobial properties of *Kalanchoe pinnata* extract, bioactive compounds content and its mode of action against pathogenic microorganisms were studied with the aim of further exploration as an alternative medicine to current synthetic antibacterial drugs [76].

The phytochemical constituents of ethanol extract of *Sida acuta* leaves and their effects on pathogenic *Escherichia coli*, *Staphylococcus aureus*, *Pseudomonas aeruginosa* and *Bacillus subtilis* bacterial species were determined using commercial ciprofloxacin as a control. The screening revealed that ethanol extract of *S. acuta* leaves possessed secondary metabolites such as alkaloids, flavonoids, phenols, tannins, terpenoids, glycosides, and cardiac glycosides. The extract exhibited significant inhibitory effects against the test bacteria. The highest antibacterial activity was exhibited at the highest concentration of the extract (300 mg/mL). The minimum inhibitory concentrations (MIC) and minimum bactericidal concentrations (MBC) were found at 37.5 and 75 mg/mL, respectively, against all reference isolates of the test bacteria [77].

Nasution et al. [78] aimed to discover the potential of herbal plants as natural antibiotic candidates by a machine learning approach based on traditional the Indonesian herbal medicine system called Jamu (Jamu is a mixture of several herbs). The input data consisted of a list of herbal formulas with their plant constituents. The target class corresponded to bacterial diseases that can be cured by herbal formulations. The best models were those implementing a random forest (RF) algorithm. This study showed that 14 plants can be potentially used as natural antibiotic candidates. Furthermore, according to scientific journals, 10 of the 14 selected plants have direct or indirect antibacterial activity.

The screening of the antimicrobial activity of *Camellia japonica flower* extracts demonstrated their potential as antibacterial agents with promising applications in the pharmaceutical and food industries. Extracts were obtained using 60% methanol as a solvent and both conventional and cost-effective maceration methods (50 °C, 1 h). The results of an agar diffusion assay revealed significant antimicrobial activity against *S. aureus* (10.29 mm), *P. aeruginosa* (9.24 mm), and *Salmonella enteritidis* (6.95 mm). However, the extracts did not exhibit activity against *E. coli*, *S. epidermidis*, and *B. cereus*, unlike other varieties of *C. japonica* that displayed activity against these microorganisms [79].

The composition, antioxidant, antimicrobial and cytotoxic characteristics of methanol and ethanol extracts from leaves of the plants *Juniperus sabina* and *Ferula communis* (Cyprus) were reported in the year 2023. Total phenolic and flavonoid content of the methanol and ethanol extracts were quantified. Mome inositol was the predominant component in *J. Sabina*’s extracts. The most dominant component of *F. communis* ethanol extract was phytol, while in the methanol extract it was 1,3,4,5-tetrahydroxycyclohexanecarboxylic acid. Antioxidant activity (by DPPH free radical-scavenging ability) testing revealed concentration dependent activity for methanol and ethanol extracts from the plant leaves. Sufficient antibacterial activity (by disk diffusion and minimal inhibitory concentration methods) of the plant extracts was found against Gram-negative and Gram-positive bacteria [80].

The shrubby plant *Opuntia stricta* is known to have medicinal properties due to its phytochemical composition. The impact of different solvents (80% ethanol, 80% methanol, and 80% acetone (*v*/*v*) in water) on extraction yield, phenolic composition, in vitro antioxidant and antibacterial activities of deseeded *O. stricta* fruit was investigated_._ It appeared that the extraction solvents significantly influenced the total phenolic content (TPC), total flavonoid content (TFC), and antioxidant capacity. The percentage of 80% ethanol shows the highest percentage for extraction yield, while 80% acetone extract shows the lowest extraction yield but the highest TPC, TFC, and antioxidant activity. The antibacterial tests demonstrated that both 80% methanol and 80% acetone extracts exhibit the highest inhibition zones (of 11.66 and 11.33 mm respectively, *p* > 0.05) against *Salmonella thyphimurium*. The 80% acetone extract demonstrated the best inhibitory effect against *Escherichia coli* (inhibition zone of 11 cm). Less sensitivity to all *O. stricta* extracts was observed in Gram-positive bacteria than in Gram-negative bacteria [81].

The bioactive components and phytochemicals of the methanol extract of *Rhanterium epapposum oliv*. (locally known as Al-Arfaj), belonging to the family *Asteraceae*, were studied using Agilent gas chromatography-mass spectrometry (GC-MS). The methanol extract of *R. epapposum* aerial parts showed the presence of sixteen compounds. The determined phytochemicals in this extract were saponins, flavonoids, and phenolic compounds. A quantitative analysis revealed the presence of high content of flavonoids, total phenolic, and tannins. These data made possible use of *R. epapposum* aerial parts as an herbal remedy for various diseases [69].

It has been reported that extracts from some *endemic plants* demonstrate different antibacterial activity against Gram-negative and Gram-positive bacteria. For example, the antibacterial potential of methanol extracts from three species of *Acantholimon* family endemic to Iran (*Acantholimon austroiranicum Rech.f. & Schiman-Czeika*, *Acantholimon serotinum Rech.f. & Schiman-Czeika* and *Acantholimon chlorostegium Rech.f. & Schiman-Czeika*) was reported to be significantly higher toward Gram-negative bacteria (*E. coli* and *P. aeruginosa)* than against *Enterococcus faecalis* and *S. aureus* [82].

Methanol and macerated methanol extracts of *Anabasis aretioides*, a plant endemic to Morocco and Algeria widely used in traditional medicine, demonstrated antimicrobial activity against *Proteus mirabilis*, *Bacillus subtilis*, *S. aureus*, and *P. aeruginosa* [83].

Good antibacterial activity of extracts and oils of *Doronicum macrolepis*, a plant endemic to Turkey, was found to be due to the high phenolic content of ethyl acetate extract. The essential oil showed an inhibitory effect on *E. coli*, *S. epidermidis*, *Enterococcus faecium*, *Yersinia pseudotuberculosis*, *C. albicans* and *C. tropicalis* [84].

Özcan et al. [84] investigated the administration of inhalable liposomes loaded with *licorice* extract (*Glycyrrhiza glabra*) for the treatment of tuberculosis. In vivo lung deposition studies of liposomal dry powder for inhalation (LDPI) in mice showed that nearly 46% of the administered drug reached the lungs and 16% of the administered drug remained in the lungs after 24 h of administration. In vivo pharmacodynamic evaluation of the LDPI against *Mycobacterium tuberculosis* showed a significant reduction of bacterial counts in the lungs and spleen. *Ginkgo biloba L*. extract was also encapsulated in nanoparticles and showed good bioavailability [85].

#### 3.1.2. Essential Oils and Fatty Acids

Several publications have highlighted the antimicrobial activity of essential oils (EOs) and their chemical constituents, including against drug-resistant microbial pathogens. Regarding the mode of action, it was shown that EOs primarily destabilize the cellular architecture of bacterial pathogens that lead to breakdown of membrane integrity and disruption of many cellular activities, including energy production and membrane transport. Membrane rupture induced by essential oils can lead to leakage of cellular components and loss of ions [86]. Among 250 reported commercially available essential oils, about a dozen have been shown to possess high antimicrobial potential, and the antimicrobial activities of the EOs were not pre-eminent for all strains. Therefore, further investigations should be focused on the targeting of EOs and microorganisms [87].

Terpenes and their derivatives comprising hydrocarbons that are usually found in EOs were reported as having potential antimicrobial activity, exhibiting bacteriostatic or bactericidal effects against test pathogens; and possible mechanisms exerted by each terpene class were discussed in [88]. The predominant antibacterial mechanism of action exhibited by EOs derived from polyphenol- and terpene-rich plants (such as *Cuminum cyminum*, *Mentha piperita*, *Thymus daenensis*, *Pimenta dioica*, *Myrtus communis*), is the disruption of the membrane function and the structure of bacterial cells and others. EOs, particularly those derived from the *Lamiaceae* and *Verbenaceae* families commonly found in the Mediterranean region, exhibit anti-quorum sensing and anti-biofilm properties against bacterial pathogens. Moreover, secondary metabolites could interfere with intermediary metabolism, as well as to disrupt DNA/RNA synthesis and functionality, and modulate critical events within the pathogenic progression [89].

The antibacterial activity of fatty acids (FAs) is accepted as a promising option for developing next-generation broad-spectrum antibacterial agents. Originating from the defense system of living organisms, FAs combined with other antibacterial agents usually demonstrate a remarkable ability to enhance their bactericidal properties. In vitro testing of the antibacterial activity of eight plant oils (nonhydrolyzed and hydrolyzed) containing medium-chain fatty acids (palm, red palm, palm kernel, coconut, babassu, murumuru, tucuma, and cuphea oil) against Gram-positive pathogenic bacteria clearly demonstrated the selective effect of the hydrolyzed forms of tested oils. While the hydrolyzed oils were active against all tested bacteria (*Clostridium perfringens*, *Enterococcus cecorum*, *Listeria monocytogenes*, and *S. aureus*) the same oils did not show any effect on commensal bacteria (*Bifidobacterium* spp. and *Lactobacillus* spp.). Tucuma and Cuphea seed oils showed the strongest antibacterial activity. Non-hydrolyzed forms of all tested oils exerted no antibacterial effect against any test bacteria. The study created a basis for the development of selective bacterial inhibitors [90].

The profile analysis (GC-MS) of FAs of tengkawang plant (*Shorea Sumatrana*) oil indicated the presence of palmitic acid, stearic acid, oleic acid, oleic acid chloride, stearic acid chloride, glycidyl stearate, diethyl phthalate, and 2-monopalmitin with predominance of stearic acid (60.68%). The diameter of the inhibition zone against *S. enteritidis*, *E. coli*, *S. aureus*, and *B. cereus* at a concentration of 12.5%, 25%, 50% (by disc diffusion test and tetracycline as a positive control), showed that tengkawang oil is an antibacterial agent with concentration optimum at 25% with more susceptibility to Gram-positive than Gram-negative bacteria [91]. The study of antibacterial properties of FAs and how their chemical structures influence the antibacterial activity aimed to better understand both the traditional and non-traditional mechanisms involved in the antibacterial activities of FAs [92]. Omega-3 fatty acids, which offer enormous nutritional benefits, were shown as therapeutically significant in treating several infectious diseases. To avoid the undesirable odor and flavor, heavy metal contamination, and extinction of fish species of fish produced FAs, oleaginous microorganisms were studied as a promising alternative to produce a more sustainable, consistent, and quality production of Omega-3 fatty acids [93]. Unsaturated FAs acting as vancomycin adjuvants were reported to rapidly kill a range of Gram-positive bacteria, including vancomycin-tolerant and resistant populations (*S. aureus* and other Gram-positive bacteria). Synergistic bactericidal activity relied on the accumulation of membrane-bound cell wall intermediates that generate large fluid patches in the membrane leading to protein delocalization, aberrant septal formation, and loss of membrane integrity [94].

Despite their promising antibacterial activity, some plant products have drawbacks that limit their therapeutic use. For instance, allicin, berberine, curcumin, emodin, linalool, oleanolic acid, quercetin, and thymol are poorly bioavailable when administered as single-compound therapies. Other disadvantages of plant-derived compounds are high volatility (e.g., linalool), low chemical stability (e.g., quercetin), pungent odor (e.g., allicin), and toxicity (e.g., sanguinarine). Structural modifications and/or loading them in drug delivery systems are two of the possible ways to avoid such drawbacks [47].

#### 3.1.3. Propolis and Honey

Resins, waxes, polyphenols, polysaccharides, volatile materials, and secondary metabolites present in the composition of propolis are responsible for its bioactivities including antibacterial. These compounds positively modulate the antimicrobial resistance of multidrug-resistant bacteria. Published research indicates that propolis and its derivatives contain many natural antimicrobial compounds that enhance the efficacy of conventional antibiotics. The activity of propolis strongly depends on seasonal and regional factors. In combinations with honey, propolis demonstrates a synergistic effect against *E. coli* and *S. aureus*. Clinical trials are necessary to better elucidate the potential application of propolis and its main flavonoid ingredients in various medical fields [95].

For centuries, honey has been used as a natural remedy to several types of illnesses and to treat wounds. Honey is known as very effective in almost all cases of infections and in the improvement of healing, especially for burn injury and wounds. Periodically, reviews have covered the antibacterial activity of honey, its use in the treatment of infections and diseases as well as the features relevant to its activity [96,97]. The antibacterial activity of honey is ascribed to the presence of various components (such as sugars, polyphenol compounds, hydrogen peroxide, 1,2-dicarbonyl compounds, and bee defensin-1) at different concentrations (depending on the source of nectar, bee type, and storage) that work synergistically. The effectiveness of honey depends on its botanical origin, the health of the bee, and the processing method. The application of antibiotics with honey yields better antimicrobial potential. In medicine, honey was used in the treatment of surface wounds, burns, and inflammation, and demonstrated a synergistic effect when applied with antibiotics. Tissue repair is enhanced by the low pH of honey (3.5–4), that causes a reduction in protease activity on the wound site, elevating oxygen release from hemoglobin and stimulating fibroblast and macrophage activity. Furthermore, H_2_O_2_ has antiseptic effects, and it disinfects the wound site and stimulates production of vascular endothelial growth. Honey can promote fast healing, reduces scarring, and is very convenient for plastic surgery. In non-infected areas, honey reduces pain and inflammation. In general, the use of honey in medical settings could reduce economic loss and provide economic benefits by lowering direct costs in comparison to conventional treatments and by using less antibiotics, faster healing, and less hospitalization stay [98]. Honey contains reactive oxygen species that are naturally produced by the body of flying insects and destroys bacteria. However, honey is sticky and difficult to apply in the correct dose. Therefore, the research team of Sophie Cox worked on ways to deliver a sterile honey product to wounds, including spray, cream, and powder making it effective in surgery, war zones and potentially in all our homes [99]. 

#### 3.1.4. Plant-Derived Antibacterial Compounds

Plants have two major groups of metabolites: primary and secondary. Carbohydrates and lipids are products of the primary metabolism while phenolic compounds, carotenoids, alkaloids, saponins, and terpenoids are considered secondary metabolites [67]. Numerous plant-derived chemical compounds with significant antibacterial activity have already been identified, belonging to different classes: triterpenoids, alkaloids, phenols, flavonoids, and large group antimicrobial peptides (defensins, cathelicidins, cecropins, melittin, thionins, nisin, and micatin) [89]. In most cases, the bioactive plant extracts contain complex mixtures of these groups, and their combined action can yield an enhanced effect [67]. For example, a comparative metabolite analysis of organs of *Piper sarmentosum* (*Piperaceae*), a traditional medicinal and food plant widely distributed in the tropical and subtropical regions of Asia, indicated 154 tentatively identified metabolites with a predominant occurrence of flavonoids, lignans, and phenylpropanoids in the leaves, aporphines in the stems, piperamides in the fruits, and lignan-amides in the roots. These extensive data on the metabolite composition of *P. sarmentosum* supplied useful information for discovery of bioactive compounds and patterns of their preferential biosynthesis or storage in specific organs. They can be used to optimize the production and to maximize the plant’s economic value as herbal medicine [100].

**Figure 4:**
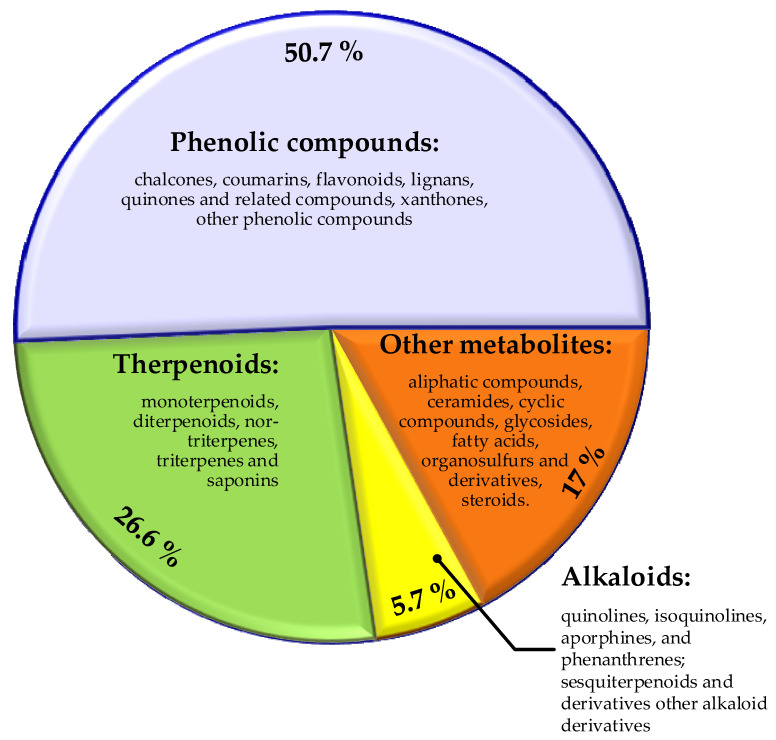
Plant derived antibacterial compounds: phenolic compounds; therpenoids, alkaloids and other metabolites.

Plant-derived compounds possess many interesting biological properties combined with multiple antimicrobial activity [101,102]. The antibacterial properties of medical plant-derived substances, such as alkaloids, tannins, flavonoids, and peptides, have been periodically discussed as a base for development of new medicines [60]. A systematic literature review of plant-derived compounds with experimentally proved antibacterial activity for the period from 2012 to 2019 showed that of 459 compounds, 50.8% were phenolic derivatives, 26.6% were terpenoids, 5.7% were alkaloids, and 17% were classified as other metabolites. Only 10 of these compounds have appeared in clinical trials [47]. The major chemical classes and sub-classes antibacterial plant-derived natural products are presented in Figure 4.

According to the current literature review, major phytochemical classes with potent antibacterial activity include phenolic compounds, alkaloids, saponins, terpenoids and others [89].

##### Phenolic Compounds

**Figure 5:**
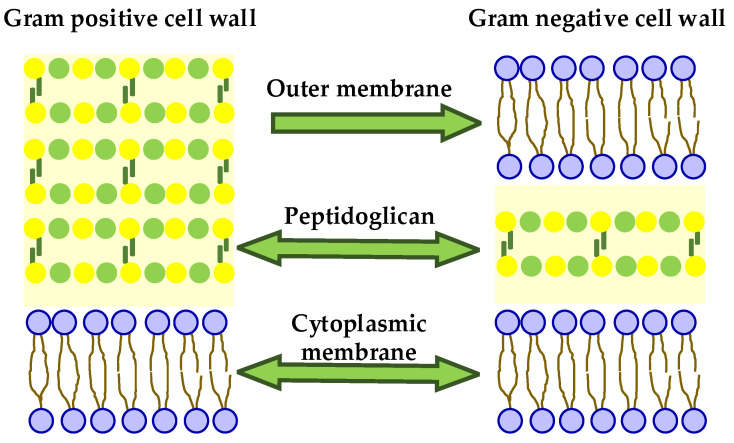
Main structural difference between the cell wall of Gram-negative and Gram-positive bacteria: Gram-negative cell wall has outer membrane, whereas Gram-positive cell wall does not contain such.

More than 8000 bioactive phenolic compounds are now known, including phenolic acids and aldehydes, flavonoids, chalcones, benzophenones, xanthones, stilbenes, benzoquinones, and polyphenols, among others, which can be extracted from different parts of the plant, such as the leaves, roots, and fruits (bark and seeds). Experimentally it was found that these compounds are more effective against Gram-positive bacteria. This could be explained by the presence of a thick peptidoglycan layer and the absence of an external membrane in them. Such outer membrane presents in Gram-negative bacteria (Figure 5) and exerts a hydrophobic action, preventing the penetration of hydrophilic molecules, such as phenolic compounds into the bacterial cells.

The main mechanism of action of phenolic compounds is associated with their ability to reduce the expression of efflux pumps. However, there are molecules, like tannins and anthraquinones that have been reported to inhibit DNA gyrase (and, thus, capable of inhibiting microbial growth) [89].

Resveratrol is a plant compound that tends to be concentrated mostly in the skins and seeds of grapes and berries. It acts as a polyphenolic antioxidant receiving attention for its potential antibacterial, anti-carcinogenic and anti-aging properties. Resveratrol displays in vitro antimicrobial activity against a surprisingly wide range of bacterial, viral, and fungal species. In combination with conventional antibiotics, resveratrol enhances the activity of aminoglycosides against *Staphylococcus aureus* [103]. Phenolic compounds from extracts of *Hibiscus acetosella* were reported to inhibit the growth of *S. aureus*, in addition to its efficiency against *P. aeruginosa* growth [104]. Prophylactic efficacy against bacterial infections by driving phagocyte influx was found for a non-bactericidal cathelicidin [105]. The available information on different types of plant phenolic compounds was systematized with emphasis on their extraction, analysis and potential biological activity [106].

Novel cathelicidin from *Hydrophis cyanocinctus* with antimicrobial and anti-inflammatory activity was identified and characterized in the year 2023 [107]. The instability of phenolic compounds to light and oxygen raises some difficulties for their extraction that could be avoided by use of adequate modern extraction, concentration, and separation technologies [108].

Flavonoids are a family of widely distributed natural phenolic compounds, produced as secondary metabolites of plants in response to diverse biotic and abiotic factors. Some of the highest amounts of flavonoids are in berries, apples, citrus fruit (oranges, lemons, grapes), spinach, legumes, kale, broccoli, soybeans, onions, tea, and cocoa [109,110]. The family of the flavonoid includes more than 6000 low molecular weight phenolic compounds that are derivatives of flavans. Flavonoids exhibiting strong antibacterial, anti-inflammatory, antioxidant, antiplatelet and other effects have been reported [111]. In some cases, flavonoids (especially chalcones) have shown up to a six-fold stronger antibacterial activity than standard drugs on the market. Some synthetic derivatives of flavonoids have also exhibited remarkable antibacterial activities against multidrug-resistant Gram-negative and Gram-positive bacteria (including *E. coli*, *P. aeruginosa* and *S. aureus*), from 20- to 80-fold higher than the activity of standard drugs [50,112]. in some cases, the naturally occurring flavonoid trans-cinnamaldehyde and its derivatives demonstrated improved antibacterial activity compared to that of commonly used antibiotics [113].

Chalcones are a class of flavonoids known as having antibacterial, anti-inflammatory, antifungal, antioxidant, cytotoxic, antitumor, and chemo preventive activities. The largest number of natural chalcones was isolated from a species of the *Leguminosae*, *Asteraceae* and *Moraceae* families [114]. Chalcones, containing α,β-unsaturated ketone fragments, are accepted as an important pharmacologically active agent because of their diverse mechanisms of antibacterial action. The relationship between the structure, biological activity, and action mechanisms provides some important guidance for the development of more candidate antibacterial agents [115].

Progress in the development of environmentally friendly methods for extraction of flavonoids from plant materials was presented. It included ultrasound, pressurized liquid extraction, mechano-chemical, high hydrostatic pressure, supercritical fluid, negative pressure cavitation, intensification of vaporization by decompression to the vacuum, microwave, infrared, pulsed electric field, high-voltage electrical discharges, deep eutectic solvents, and enzyme-assisted extraction, as well as the impact of the operation conditions [116,117]. The current advanced technologies for flavonoids extraction from natural sources are faster, more environmentally friendly, and with higher automation levels, compared to conventional extraction techniques [118].

Experimental design combined with modeling could reduce the number of experiments that should be performed to achieve maximum extraction yield [119]. Based on the single factor experiment and Plackett–Burman design results, the microwave-assisted extraction of flavonoids from *Phyllostachys heterocycla* leaves was further optimized, using the response surface methodology, [120]. An investigation of the effects on total flavonoid content, flavonoid composition, and stability in jujube extracts was prepared by five extraction methods: (i) water–water bath, (ii) ethanol–water bath, (iii) deep eutectic solvent, combined with ultrasound-assisted extraction (DES-UAE), (iv) microwave-assisted extraction, and (v) enzyme-assisted extraction It was demonstrated that DES-UAE was the most efficient method for flavonoid extraction from jujube [121]. The effect of extraction solvents (hexane, ethyl acetate and methanol) on phenolic compounds and flavonoids from different parts of pongame oil tree (*Derris indica*) (that could be a potential source to develop pharmaceutical products) against bacterial pathogens demonstrated that the ethyl acetate extract had the highest content of phenolic compounds and flavonoids, [122]. An analysis of flavonoid compounds isolated from the leaves of Akalifa (*Acalypha wilkesiana Muell. Arc.*) by maceration and methanol solvent extraction, addition of ethyl acetate, and partitioning with n-hexane proved that the flavonoid is an isoflavone [123].

##### Alkaloids

More than 12,000 alkaloid compounds isolated from plant extracts are already known to have medicinal actions, such as antibacterial, antitumor, and analgesic (morphine and codeine) properties [124]. Alkaloids have a chemical structure with heterocyclic rings containing N-heterocyclic nitrogen and could be classified according to their carbon precursors and structure. Examples of alkaloid compounds, commonly found in plants include pyridine, piperidine, quinoline, alkaloidal amines, and terpenoids [89]. Tryptanthrin is a natural alkaloid with indoloquinazoline content. This alkaloid could be synthesized chemically and could be derived from natural sources like plant extracts or cell cultures, including yeast. It has good anti-cryptococcal activity and has a synergistic effect in combination with calcineurin inhibitors in vitro, though it is less expressed in vivo due to a poor blood–brain barrier penetration [125]. Good antimicrobial action of benzyl tetrahydroisoquinolin alkaloids, derived from the leaves of *Doryphora aromatica* was demonstrated against methicillin resistant isolates of *Mycobacteria* spp. and *S. aureus* [126].

##### Terpenoids

Terpenoids, or terpenes, are a class of metabolites that encompass a variety of natural substances, which have in common the presence of C5 isoprene units in their chemical structure. Depending on the amount of C5 isoprene involved in their synthesis, terpenes are monoterpenoids, sesquiterpenoids, diterpenoids, sesterterpenoids, and triterpenoids. More than 40,000 terpenoid substances are known with different applications, including pharmaceutical, aromatic, agricultural, and industrial [89]. The antibacterial action of terpene compounds from *Eremophila lucida* was demonstrated against *S. aureus* isolates [127]. Terpenoids from *Commiphora resin* were isolated and identified with good antibacterial action against sensitive and resistant isolates of *Mycobacterium tuberculosis*, [128]. Andrographolide, a terpene derivative from *Andrographis paniculata*, was reported as able to inhibit invasive microbe virulence factors and regulate the host immunity. Controlled clinical trials revealed that *A. paniculata* treatment was safe and efficient against acute respiratory tract infections like common cold and sinusitis. Therefore, it was considered that *A. paniculata* and andrographolide, could be accepted as excellent candidates for antimicrobial drug development [129]. Pech-Puch et al. [130] verified the good (MIC in the range of 1–8 µg/mL) and moderate (MIC value of 16 µg/mL) antimicrobial action of diterpene alkaloids from *Agelas citrina* against the Gram-positive pathogens *S. aureus*, *S. pneumoniae*, and *E. faecalis* and the Gram-negative pathogens *A. baumannii*, *P. aeruginosa*, and *K. pneumoniae*.

Saponins have been found in a variety of plants. They are chemically characterized by the presence of glycosylated groups, formed by a hydrophilic and a lipophilic part. This structure confers detergent and surfactant properties of saponins [67]. It is known that the chemical structure of saponins directly interferes with the effectiveness of their antimicrobial action. Saponins with tri-saccharide chains exhibited good antifungal action, whereas saponins with mono- or di-saccharide chains did not show good antimicrobial action [131].

##### Other Compounds

Lipids (essential oils, fixed oils, sterols, waxes, phospholipids, and fat-soluble vitamins) are another class of naturally occurring compounds. They are categorized as primary metabolites; nevertheless studies have shown that they have secondary metabolite functions [132].

The pelargonic acid micelles, extracted from tomatoes, were found to demonstrate antimicrobial efficacy against *Salmonella* that varies by the surfactant, strain serotype and stress response [133]. A recently published report confirmed the antibacterial activity of garlic and onions, exhibiting inhibitory effects on diverse bacteria. The inhibitory effect was due to their abundant sulfoxide contents imparting them with antimicrobial properties. On the other hand, horseradish, mustard seeds, and wasabi demonstrated inhibition activity that was attributed to their elevated levels of allyl glucosinolates [89].

Seven bioactive compounds (berberine, catechin, chelerythrine, cinnamaldehyde, ellagic acid, proanthocyanidin, and sanguinarine) originating from plants were recently presented with antibacterial activity against *Staphylococcus aureus*, *Enterococcus* spp., *Klebsiella pneumoniae*, *Acinetobacter baumannii*, *Escherichia coli*, *Serratia marcescens* and *Pseudomonas aeruginosa*, as natural antimicrobial agents for the treatment of wound infections [134].

It is thought that the utilization of plant-derived phenolic compounds, alkaloids, saponins, and terpenoids as effective antibacterial drugs could be optimized by omics technologies and network pharmacology to identify optimal combinations among these compounds or in conjunction with antibiotics [89].

##### Possible Antibacterial Modes of Action of Plant-Derived Antibacterial Agents

Most natural antibacterial agents appear to impact the bacterial membrane permeability, leading to membrane rupture and cell lysis. However, not all mechanisms of action were elucidated and sometimes the mechanism may be indirect, stimulating the host immune system or inhibiting adhesion to the host cell. Due to the structural differences between Gram-negative and Gram-positive bacteria (Figure 5) the efficacy of antimicrobial agents varies [135,136].

**Figure 6:**
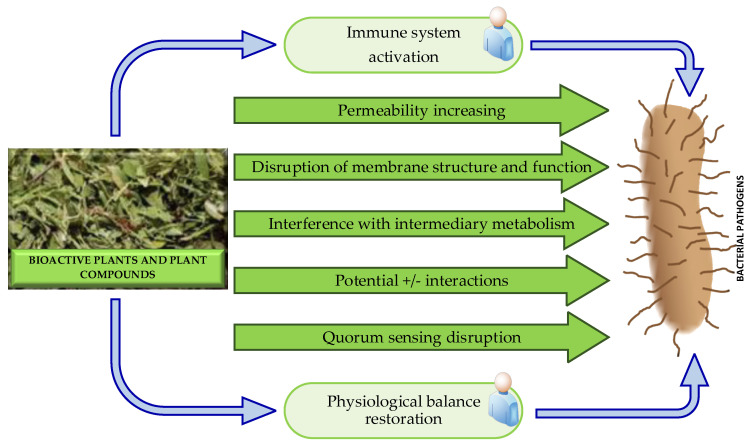
Possible mode of antibacterial action of medicinal plants: increasing the permeability; disruption of membrane structure and function; interference with intermediary metabolism; potential; plus/minus interactions; quorum sensing disruption; immune system activation; and physiological balance restoration.

Generally, the antibacterial properties of medicinal plants are hypothesized to be connected to two mechanisms: chemical interference with the synthesis or functioning of most important bacterial components, and/or bypassing the conventional mechanisms of antibacterial resistance. The possible antibacterial modes of action of plant-derived antibacterial agents are illustrated in Figure 6.

Modes of antibacterial action include the immune system activation, increased permeability, disruption of the membrane function and structure, interference with the intermediary metabolism, potential alternation, anti-quorum sensing and anti-biofilm activity, and restored physiological balance. The latter is a holistic mechanism that is totally absent in modern antibiotic treatments. Plants may alter the physiological balance of the body and make it more resistant to pathogens, whereas in modern medicine, drugs are in a form of single bioactive compounds that are designed to target a specific disorder or infection. A general feature of traditional medicine is synergism, which provides multiple targets against specific diseases. More detailed information about the mechanisms of action of plant-derived antibacterial agents could be found in [89].

### 3.2. Antibacterial Peptides Derived from Terrestrial Biota

**Figure 7:**
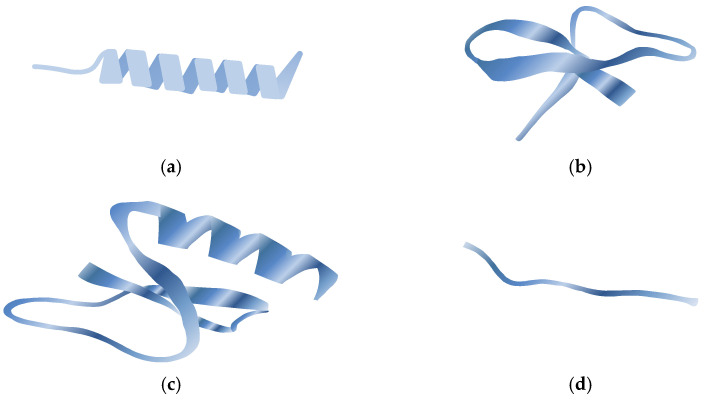
Secondary structure of antibacterial peptides: (**a**) α-helix, (**b**) β-sheet, (**c**) loop, and (**d**) extended.

Natural antibacterial peptides (ABPs) are low-molecular-weight peptides obtained by animals, plants, and microbials, where they act as a significant part of the innate immune system and demonstrate a broad range of antimicrobial and immunomodulatory effects against bacteria. Natural ABPs are positively charged and usually contain fewer amino acids [137]. The large diversity of natural ABPs makes their classification challenging. Nevertheless, they are categorized according to the origin as either plant-derived, animals-derived, or microbial-derived; according to activity as either antibacterial, antifungal, antiviral, antiparasitic, or antitumor; and according to the secondary structure as either β-sheet, α-helix, loops or extended peptides. The secondary structure of AMPs: α-helix, β-sheet, loop and extended is depicted in Figure 7. The majority of AMPs belong to the β-sheet and α-helix groups [42,137,138,139,140].

Natural ABPs have numerous advantages compared to conventional antibiotics that explain the high interest for their utilization in bacterial therapy, including multidrug-resistant infections [141,142]. They are effective at low concentrations and against certain types of bacteria that are resistant to common antibiotics such as vancomycin-resistant *Enterococcus* and methicillin resistant *Staphylococcus aureus*, and others. Furthermore, AMPs in combination with conventional antibiotics demonstrate a synergistic effect. They are considered safe for use, with less or lack of side effects. As an added benefit, they possess broad-spectrum antimicrobial properties as compared to traditional antibiotics [143,144], high sensitivity [145], and multifunctional activity, i.e., they are able to target several points of interest [146]. Many ABPs can directly kill pathogenic microbes, whereas some operate indirectly by regulating various host defensive systems. In addition to direct bactericidal action, several AMPs exhibit complex immunomodulatory activity thereby indirectly promoting pathogen purification of the host [147]. The immunomodulatory functions of ABPs include the following: enhanced chemotaxis of immune cells activation and differentiation, including dendritic cell maturation and initiation of adaptive immunity; repression of cytokine-mediated and Toll-like receptor (TLR)-mediated release of pro-inflammatory cytokines and reactive oxygen species; induction of anti-inflammatory cytokines; scavenging of bacterial endotoxins, stimulating angiogenesis, enhancing wound healing, and reducing scar formation [148]. Most ABPs exert the following:

(i) direct biocidal effect by disrupting the membrane integrity of the target organism and/or by translocating across the microbial membrane to reach the intracellular targets;
(ii) membrane interactions, mediated by the electrostatic forces between the positively charged ABPs and negatively charged bacterial surface;
(iii) destabilization of the bacterial membrane by decreasing or increasing its thickness or by causing clusterization of phospholipid head groups in the membrane;
(iv) affecting membrane permeabilization by formation of a complex with small organic anions carrying them across the membrane [149,150].

The low ability to develop drug resistance and the high antibacterial activity of ABPs are explained to some extent by possible mechanisms of their action that could be simplified and summarized as including: (1) Inhibition of NRA/protein synthesis; (2) Degradation of DNA; (3) Ion-permeable pore formation in the cell membrane; and (4) Peptidoglycan synthesis inhibition [43,150].

In contrast to antibiotics, ABPs realize contact with the cell membranes by neutralizing the charge, and then pass through the membranes to destroy the bacteria and lessen the likelihood those will develop drug resistance [143]. Since ABPs have antimicrobial mechanisms separate from conventional antibiotics, they are accepted as one of the best choices for the treatment of pathogenic and drug-resistant bacterial infections [137].

The distribution of ABPs among virtually all living organisms, complemented by exceptional structural and functional variety, stipulates a range of different antimicrobial activities including the development of new antibiotic lead molecules [151,152].

The first antimicrobial peptide (AMP) was discovered in 1957 by Robert Skarns in blood cells [146]. The key advantages of ABPs include slow emergence of resistance, broad-spectrum antibacterial activity, high sensitivity, ability to modulate the host immune response. They have rapidly gained attention, and now ABPs are accepted as promising next-generation antibiotics that can be used to combat drug-resistant pathogens [141,142].

From the large amount of ABPs discovered until 2020, the FDA approved seven: gramicidin, daptomycin, colistin, vancomycin, oritavancin, dalbavancin, and telavancin. Analyses of the FDA approved drugs database demonstrates that all FDA-approved ABPs were found in Gram-positive soil bacteria, and 98% of the known ABPs come from natural sources (skin secretions of frogs and toxins from different species) [147].

Encouraging examples of several ABPs already introduced into medical practice exist, such as the well-characterized cyclic anti-infective peptides gramicidin and polymyxins. Numerous other ABPs were under evaluation in non-clinical settings and in clinical trials up to 2020. Examples of ABPs that have progressed into late-stage clinical studies include the following: omiganan, pexiganan, and DPK060, developed for the treatment of bacterial infections; LL-37, developed for improved healing of venous leg ulcers; and PXL01, evaluated for preventing postsurgical adhesions. These ABPs are linear, cationic and have a molecular length varying from 12 (omiganan) to 37 (LL-37) amino acids [150].

Up to the year 2022, more than 1500 natural AMPs were identified and some of them like Novexatin, Omiganan, Pexiganan, thionins, and thioninetic are passing or passed preclinical or clinical trials, [43].

In 2023, the size of the market of natural antimicrobial peptides was evaluated at USD 223.00 Mn. It is expected this market to reach USD 532.02 Mn by the year 2031 and to increase by 11.65% during the forecast period 2024–2031 [3].

Up to 2024, only a small number of the thousands of discovered ABPs were approved by the FDA for clinical use. Because of their extremely short half-lives, they were recommended only for intravenous administration, topical treatments and, in certain cases, oral administration in the form of a very small tablet or capsule formulation. The FDA approved ABP formulations are as follows: Atazanavir, Boceprevir, Bulevirtide, Colistin, Dalbavancin, Daptomycin, Enfuvirtide, Glecaprevir, Indinavir, Lopinavir, Nelfinavir, Oritavancin, Oseltamivir, Peramivir, Remdesivir, Ritonavir, Saquinavir, Teicoplanin, Telaprevir, Telavancin, Tesamorelin, and Vancomycin. Some ABP-based formulations as drugs currently in clinical trials include the following: Murepavadin, EA 230, Ghrelin, SGX942, p2TA (AB103), PMX-30063 (Brilacidin), hLF1–11 (Fungal infections), Friulimicin B, PLG0206, IDR1, Omiganan (MBI-226), Pexiganan (MSI-78), LTX-109, OP-145, DPK-060, NP101 and NP108, Novexatin (NP213), P113 (PAC-113), SGX942; Iseganan (IB-367), Brilacidin (PMX-30063), and Ctx (Ile21)-Ha [137].

The discovery of novel antibiotics using natural ABPs remains a significant current challenge. Some drawbacks, like metabolic instability and/or toxicity, difficulties in the design for specific targets and others, make their clinical application problematic. Structural and functional limitations, combined with strict environmental regulations hamper the clinical translation of antimicrobial peptides as potential therapeutic agents [145].

#### 3.2.1. Plant Antibacterial Peptides

Plant antimicrobial peptides (ABPs) that play an important role in their innate immunity have broad-spectrum antibacterial activity, rapid killing, and cell selectivity, [153]. Primary plant ABPs target cell membranes or intracellular components in a variety of ways, which enables them to effectively kill a wide range of microorganisms and reduce the chance of pathogens to develop resistance [154,155].

Plant ABPs have advantages compared with current antibiotic drugs because they possess a naturally occurring defense mechanism used by plants since antiquity in fighting pathogenic bacteria. Plant ABPs are underutilized, and their products offer great promise as a novel source of drug discovery for treating human infections and other diseases to solve myriad problems confounding pathogen resistance and lack of antibiotics sensitivity [156]. Their potential applicability in bacterial disease treatments gather more and more interest. Molecular-based delivery, classification, production, mode of action, and chemical synthesis have shown a growing popularity over the years, [157]. Extraction methods are used for isolation of ABPs from plant organs, directed to obtain some specific structural types of ABPs [158]. Optimized methods for chemical extraction of ABPs from roots and leaves of extremophile plants *Anthyllis sericea* and *Astragalus armatus* (collected from the Tunisian desert) were described based on using sulfuric acid, dichloromethane, phosphate buffer, acetic acid, and sodium acetate as solvents. The most appropriate solvents for extraction of ABPs from both *An. sericea* and *As. armatus* appear to be acetic acid and sodium acetate. Respectively, the corresponding leaves and roots extracts demonstrate activity against Gram-positive and Gram-negative bacteria [155,156,159].

The most accepted classification of plant ABPs is based on their sequence similarity and tridimensional structures. In these respects, they are classified as thionins, defensins, hevein-like peptides, knottins, stable-like peptides, lipid transfer proteins, snakins, and cyclotides [160].

Thionins were found only in some plant families of *Angiosperms*. The One Thousand Plant Transcriptomes Initiative (1KP project) sequences the transcriptomes of more than 1000 plant species and uses the data to search for new thionin sequences. There were many hits from the *Angiosperm* plant families, which were previously not known to contain thionins. A large gene family for thionins was found in *Papaver*. A thionin encoded by a genomic clone was found that has antimicrobial activity in vitro. Previously, thionins were grouped into four classes. New data provided a reason to revise this classification. There are now recognized only class 1 thionins with eight cysteine residues and class 2 thionins with six cysteine residues [161].

Cruciferins, napins, oil-body proteins and oleosins are seed storage proteins (SSPs) in mustard and rapeseed (*Brassica napus* L., *B. juncea* L., *B. nigra* L., *B. rapa* L. and *Sinapis alba* L.), that have been used in traditional medicinal systems against different infectious diseases. Both in silico and in vitro antibacterial activity of napin and cruciferin rapeseed proteins provided a reason to be accepted as potential candidates for development of new antibacterial agents and to be used in complementary medicine to alleviate bacterial diseases, [162]. Shotgun proteomics of *Brassica rapa* seed proteins identified vicilin as a major seed storage protein in the mature seed [163].

Several bioactive ABPs including defensins (protease inhibitor), lectins, thionin-like peptides, vicilin-like peptides, and snaking were isolated from the plants of *Solanaceae* family [164,165]. Several important amino acid-derived classes were presented as plant defensive compounds, including antimicrobial peptides, defensins, thionins, and knottins as potential drug leads, examining their mechanisms of action, therapeutic targets, and structure–function relationships [166].

*Solanaceae* is an important family of flowering plants (*Angiosperms*) that comprise over 100 genera and more than 3000 species. The *Solanaceae* family plants are rich in different bioactive constituents including natural ABPs that have been used in different traditional medicinal systems. Several bioactive ABPs including defensins (protease inhibitor), lectins, thionin-like peptides, vicilin-like peptides, and snaking were isolated from the plants of the *Solanaceae* family [164,165].

The successful use of plant ABPs requires detailed knowledge of the structure–function relationship. The last one was studied in thionins, α-harpinins, hevein-like peptides, and the unique Ib-AMP peptides isolated from *Impatiens balsamina*. It was shown that even subtle changes in amino acid sequences could affect the biological activity of ABPs, which opens possibilities for the creation of molecules with better therapeutic efficacy and cheaper large-scale production [167].

There are studies reporting some toxicity of plant ABPs to non-target cells or limitations of oral administration. However, it is accepted that ABPs with reduced toxicity, allergenicity, or greater resistance to peptidases could be designed by chemical modification strategies and different bioinformatics tools [160].

It is thought that the discovery and design of ABPs with desired properties (reduced toxicity, allergenicity, or greater resistance to peptidases) could be performed by database analyses, chemical modification strategies, and different predictive bioinformatics tools [155,156].

Multiple classes of antimicrobial peptides in *Amaranthus tricolor* (red amaranth or Chinese spinach) were revealed by in silico prediction, proteomics, and mass spectrometric characterization. Bottom-up proteomics identified seven novel peptides from three ABP classes including lipid transfer proteins, snakins, and a defensin. Bioactivity screening of isolated Atr-LTP1 showed activity against the high-risk ESKAPE bacterial pathogens: *Enterococcus faecium*, *Staphylococcus aureus*, *Klebsiella pneumoniae*, *Acinetobacter baumannii*, and *Enterobacter cloacae*. The results highlighted the potential for integrating ABP prediction algorithms with complementary OMICS approaches to accelerate characterization of biologically relevant ABP forms [168].

A user-friendly web server, termed PTPAMP, available at the URL: http://www.nipgr.ac.in/PTPAMP/ (18 March 2024) was developed to be used as a prediction tool for plant-derived antimicrobial peptides by integration of selected compositional models. So far, developed models have been based on multiple peptide features, like amino acid and dipeptide composition, as well as physicochemical attributes for predicting plant-derived ABPs. The selected compositional models were integrated into the PTPAMP server, that is capable of classifying a queried peptide sequence into four functional activities: antimicrobial, antibacterial, antifungal, and antiviral. The analysis indicated the abundance of cysteine residues in plant-derived ABPs and the distribution of other residues like G, S, K, and R, which differ as per the peptide structural family [169].

Recently, recombinant ABPs were produced in plants at large scale and low cost. Because the ABPs are less likely to elicit resistance to pathogenic bacteria than conventional antibiotics, it seems that they will open new avenues for agricultural and medical applications [170].

#### 3.2.2. Animal Origin Antibacterial Peptides

Natural antibacterial peptides (ABPs) are present in every animal as a part of its innate immune system; ABPs are derived from a variety of animals including mammals, amphibians, in snake venom, crabs, insects, and others.

##### Antibacterial Peptides Derived from Mammalians

ABPs were isolated from different mammalian sources such as granules of neutrophils, Paneth cells, mucosal secretions from epithelial cells and protein degradation products. Three classes of antibacterial peptides, found in abundance in neutrophils defensins, cathelicidins and histatins were studied extensively [171].

Cathelicidins are a class of natural, short cationic ABPs identified in different animal species, such as birds, fish, reptiles, amphibians, in snake venom, and mammals (cow, pig, rabbit, sheep, mouse, monkey, horse, and human). They are primarily produced in epithelial cells, neutrophils, and macrophages. About 30 cathelicidin family members were identified in mammalian species. The cathelicidin family of ABPs with varying antibacterial activities and safety is now considered now a promising alternative to conventional antibiotics [172].

##### Antibacterial Peptides Derived from Amphibians

Amphibians are important sources of ABPs. *Duttaphrynus melanostictus* is the main source of the traditional Chinese medicine “Chansu”, which has anti-infection effect without a clear mechanism. A study aimed to find a cathelicidin peptide in *D. melanostictus*, investigated its activity in vivo and in vitro, and an AMP-encoding gene (cathelicidin-DM, GenBank: KJ820824.1) was obtained from the constructed cDNA library of *D. melanostictus*. A skin wound infection model and in vivo imaging were used for evaluation of possible applications. The results showed that cathelicidin-DM is a 37 amino acid AMP with good bactericidal ability, similar to that of melittin. Both can kill bacteria within 15 min. Thus, cathelicidin-DM could be a new template for antimicrobial drug development based on its good antibacterial activity in vivo and in vitro [173].

Another antimicrobial peptide isolated from frog skin hemocytes is poly(glycolide-co-lactide) (PGLA); it was shown to prevent bacterial adhesion by causing a conformational change and elimination of bacterial pili. One more antimicrobial peptide, magainin was isolated from the African clawed frog (*Xenopus laevis*). However, studies show that resistant *Escherichia coli* strains can easily develop resistance [23].

##### Antibacterial Peptides Derived from Snake Venom

Cathelicidins are a class of natural, short cationic AMPs identified in different animal species: mammals, birds, fish, reptiles, amphibians, and in snake venom. About 30 cathelicidin family members were identified in mammalian species. The data identified in snake venom cathelicidins, including their chemistry, characterization, pharmacological action, antimicrobial and antibioflim effects, mechanism of action, and a potential for the development of novel antibiotics to combat antibiotic-resistant bacteria were summarized by Barros et al. [172]. Cathelicidins were first studied mostly for their direct antimicrobial killing capacity, nowadays they are more and more appreciated for their immunomodulatory functions, [174]. According to Yang et al. [105], a non-bactericidal cathelicidin provided prophylactic efficacy against bacterial infection by driving phagocyte influx eLife.

Wang et al. [107] reported a newly identified cathelicidin peptide, named Hydrostatin-AMP2, from the snake *Hydrophis cyanocinctus* with excellent antimicrobial activity against both Gram-positive and Gram-negative bacteria, including standard and clinical Ampicillin-resistant strains. The newly identified Hydrostatin-AMP2 had faster antimicrobial action than Ampicillin as demonstrated by a kinetic assay. Hydrostatin-AMP2 apparently decreased the production of pro-inflammatory cytokines in the LPS-induced RAW264.7 cell model.

Klubthawee et al. [175] reported a rationally designed, hybrid antimicrobial peptide inspired by cathelicidin and aurein and exhibiting membrane-active mechanisms against *Pseudomonas aeruginosa*.

##### Antibacterial Peptides Derived from Crabs

Jiang et al. [176] reported a novel antibacterial peptide Spampcin56–86 from *Scylla paramamosain* crab exerting rapid bactericidal and anti-biofilm activity in vitro and anti-infection in vivo. Many diseases in the modern world originate from inflammatory and oxidative stress. Beneficial metabolites with promising antioxidant and anti-inflammatory potential have been recently reported, that were identified in the methanol extract from the shell of crabs of the genus Charybdis [177].

##### Antibacterial Peptides Derived from Insects and Others

Lee and Shin [178] identified novel ABPs from the venom gland of the spider *Pardosa astrigera* by deep multi-task learning.

Different types of antimicrobial agents, obtained from animals/animal products are known to demonstrate antimicrobial potency against various pathogenic microbes. Such are the following: lactoferrin (Lf), an iron binding milk protein that is active against a spectrum of microbial organisms like *E. coli*, *Carnobacterium*, *Klebsiella*, *L. monocytogenes*; chitosan, a polycationic biopolymer found naturally in the crustacean and arthropod exoskeletons that is active against various Gram-positive and Gram–negative bacteria; the bacteriolytic enzyme, lysozyme, that is found naturally in mammalian milk and hens eggs displaying an excellent antimicrobial action in the case of *Listeria innocua* and *Saccharomyces cerevisiae*; certain milk-derived bioactive substances, like casein, that are reported to have antibacterial activities and others [43].

Arenicins are a group of three types of peptides: arenicin-1, arenicin-2, and arenicin-3 that were shown to have good antimicrobial activity against Gram-negative bacteria. Arenicin-1 isolated from *Arenicola marina* (sandworm) shows potent antimicrobial activity against *Escherichia coli* and *Pseudomonas aeruginosa*, with MIC values between 1 and 2 µM. A slightly modified arenicin-3 peptide has considerable antimicrobial activity even against XDR (extensive drug resistance) and MDR (multidrug resistance) strains such as *Pseudomonas aeruginosa*, *Acinetobacter baumannii*, *Escherichia coli* and *Klebsiella pneumonia* [179].

#### 3.2.3. Antibacterial Agents Produced by Microbes

Microbial secondary metabolites are a great source of bioactive molecules. The discovery of penicillin (efficient against Gram-positive bacteria), derived from *Penicillium notatum* by Alexander Fleming, in the year 1928, made a fundamental change in the sources for derivation of natural biologically active products from plants to microbes [43,180,181]. Natural products with diverse structures and biological activity and recombinant proteins, produced by microbes, are already valuable molecules for medicine. The development of robust and promising microorganisms, such as cell factories, and engineering approaches directed to improve yields of microbial production and generating novel molecules are largely studied and are inspiring the development of new therapeutic agents [182].

Converting carbon and nitrogen sources into a large diversity of intracellular and extracellular biopolymers, such as polysaccharides, polyamides, polyesters, polyphosphates, extracellular DNA, and proteinaceous components by bacteria is also of research interest as a new target for antibacterial drugs. Bacterial polymers have important roles in pathogenicity, and their varied chemical and material properties make them suitable for medical and industrial applications. When produced by pathogenic bacteria, they function as major virulence factors; whereas when produced by non-pathogenic bacteria, they become food ingredients or biomaterials [183].

Produced by microbes, both bacteriophages and bacteriocines are two types of antibacterial agents of particular interest in combating antimicrobial resistance (AMR).

##### Bacteriophages

Bacteriophages (phages) are viruses able to infect bacterial cells and force them to produce viral components, using a lytic or lysogenic cycle. Bacteriophages are a potential alternative to chemical antimicrobial agents used against pathogens that are of public health significance. Understanding the phage diversity and host specificity is important for the development of effective phage biocontrol approaches [184].

Thousands of bacteriophages of different types can be found everywhere. Bacteriophages were discovered over a century ago, but their use for treatment of antibiotic-resistant infections regained popularity only recently. In contrast to many antibiotics, which damage harmful bacteria, and simultaneously disturb the whole microbiota thus triggering a new set of problems, each phage more narrowly targets bacterial strains or species. This specificity makes phage therapy an attractive approach for treating antibiotic-resistant bacterial infections, including such caused by multidrug-resistant bacteria. The bacteriophages provide other advantages over antibiotics, like less significant side effects, less time-consuming and less costly development process, [185,186]. Because of its many advantages, phage therapy is currently experiencing a renaissance after long years of different doubts. Recently, the U.S. National Institutes of Health (NIH) awarded $2.5 million to 12 institutes around the world to study phage therapy [187,188].

Phages are the only drug that reproduces itself at the site of infection and disintegrates again after lysis of all suitable bacteria. They contain DNA or RNA in their genome that is encapsulated in a protein coat. Many phage proteins, including endolysins (lysins), virion-associated peptidoglycan hydrolases (VAPGHs), depolymerases, and holins display antibacterial activity. Since several phage types may be suitable for one bacterial pathogen from a set of available phage types, the therapeutic use of mixtures of different phage types is optimal. The specificity of phages is their major advantage over antibiotics. It is also the main reason for the individual character of phage application as a tailor-made therapy in individual cases. Main families and characteristics of bacteriophages were thoroughly presented by Bin Hafeez et al. [139].

Phages against *Staphylococcal* Infections

In terms of potential medical applications, phages belonging to the Kayvirus genus of the Herelleviridae family are regarded as the most interesting ones. Kayviruses have already demonstrated their efficacy in the treatment of various *Staphylococcus aureus* infections, both in animal models and human clinical cases [189,190]. A phage belonging to the Kayvirus lytic module was isolated that encodes an additional endolysin [191]. The polyvalent, Kayvirus genus phages, infecting mostly *S. aureus* and some CoNS and displaying a broad-spectrum biological activity are one of the best agents controlling staphylococcal infections [192,193,194]. Some of the Kayvirus phages have already been used in commercial phage-based preparations to treat DFI [195]. Studies on phage therapy for staphylococcal infections are focused mainly on *S. aureus* and only a few on the isolation and characterization of phages infecting the clinical isolates of CoNS, especially *Staphylococcus epidermidis* [192,195].

Fanaei Pirlar et al. [196] described one of the novel bacteriophages specific against *S. epidermidis* and with anti-biofilm activity. Important for the success of phage therapy, in vitro techniques, and measurements of phage characteristics were presented [197]. A comparative assessment of the bacteriophage and antibiotic activity against multidrug-resistant *S. aureus* biofilms showed that, while the antibiotics cannot diffuse through the polymeric matrix of a biofilm, the Kayviruses can effectively penetrate and disrupt *S. aureus* and *S. epidermidis* biofilm structures [198,199].

Alsaadi et al. [200] reported the isolation and genome sequencing of 40 bacteriophages from human skin SWABS that infect coagulase-negative *Staphylococcus* (CoNS) species, which extended the knowledge of phage diversity. Six genetic clusters of phages were identified with two clusters representing novel phage species, one of which was characterized and named Alsa phages. The identified Alsa phages have a greater ability to infect the species *S. hominis* that were otherwise less infected than other CoNS species by the isolated phages. This indicates an undescribed barrier to phage infection that could be due to numerous restriction-modification systems. The extended diversity of *Staphylococcus phages* here enables further research to define their contribution to skin microbiome research and the mechanisms that limit phage infection.

*Staphylococcus* sp. is the most common bacterial genus in infections related to diabetic foot ulcers (DFUs). Plumet et al. [201] isolated six phages (SAVM01 to SAVM06) from effluents of diabetic foot ulcers (DFUs), belonging to the *Herelleviridae* family, with sequences similar to those of the *Kayvirus* genus. No lysogeny-associated genes, known virulence, or drug resistance genes were identified in the phage genomes. The phages displayed a strong lytic and anti-biofilm activity against DFU clinical isolates, as well as against opportunistic pathogenic coagulase-negative *staphylococci*. The experimental results suggest that these phages could be effective biocontrolling agents against *staphylococcal* clinical isolates from DFUs.

Phages against *Pseudomonas* Infections

Bacteria surviving in extreme conditions and the bacteriophages that infect them are sources of heat-stable proteins that are utilized in biotechnological applications but not as antimicrobial agents. Plotka et al. [202] demonstrated that the Ts2631 endolysin from the extremophilic bacteriophage vB_Tsc2631, which infects *Thermus scotoductus*, is very active against the multidrug-resistant clinical strains of *Acinetobacter baumannii*, *Pseudomonas aeruginosa* and pathogens from the *Enterobacteriaceae* family, i.e., Ts2631 endolysin could be an effective antimicrobial agent against Gram-negative multidrug-resistant bacteria. Transmission electron microscopy (TEM) and fluorescence microscopy observations of *A. baumannii* cells, treated with Ts2631 endolysin variants, demonstrated that the intrinsic antibacterial activity of Ts2631 endolysin depends on the presence of its N-terminal tail [202].

A novel phage, pPa_SNUABM_DT01, infecting *Pseudomonas aeruginosa* canine otitis externa isolates was characterized by its morphology, growth, lysis kinetics, and genomic characteristics. A comparative genome analysis demonstrated that the phage was a novel species in *Myoviridae*. The nucleotide similarity was moderately high compared to the *Pseudomonas* virus, Noxifer. However, a phylogenetic analysis and a dot plot indicated that pPa_SNUABM_DT01 was not closely related to the *Phikzvirus* or *Noxifervirus* genus, instead, it was shown to belong to a novel one [203].

*Proteus mirabilis* and *P. aeruginosa* are two bacterial species commonly associated with urinary tract infections in humans. Novel bacteriophages and their derived proteins were developed for the biocontrol of *Proteus* and *Pseudomonas* biofilms. The identification and utilization of both the whole phage and its tail-spike protein with the pectate lyase activity were described to treat *P. mirabilis* biofilms. The phage and the tail-spike protein were assessed by different in vivo and in vitro assays that demonstrated their antibacterial and antivirulence properties against *P. mirabilis* biofilms. The combination treatment of *P. aeruginosa* biofilms by phage and cold atmospheric plasma established that the use of cold atmospheric plasma followed by exposure to *P. aeruginosa* phages was the most effective for eradication of *P. aeruginosa* biofilms [204].

Two bacteriophage genera (targeting *B. mycoides* and *Pseudomonas species*) discovered in a groundwater reservoir highlighted subsurface environments as underexplored biotopes in bacteriophage ecology [205].

Phages against *Escherichia coli* Infections

Sattar et al. [206] reported isolation, preliminary characterization, and genome analysis of two novel lytic phage species (*Escherichia* phage SKA49 and *Escherichia* phage SKA64) having lysis potential against MDR strain of avian pathogenic *E. coli*, QZJM25. Both phages, SKA49 and SKA64, were able to keep QZJM25 growth significantly less than the untreated bacterial control for approximately 18 h, being stable at 37 °C only. In contrast to SKA64, SKA49 demonstrated a broader host range against *Escherichia coli* strains. Genome analysis indicated their safety because no recombination, integration and host virulence genes were identified.

Nicolas et al. [207] isolated and characterized a novel phage collection against avian-pathogenic *E. coli* (APEC). The collection includes nineteen genetically diverse, lytic *E. coli* phages, eight of which were tested in combinations for their efficacy in controlling avian pathogenic *E. coli* infections. Genome homology analysis revealed that the phages belonged to nine different genera, one of them being a novel genus (Nouzillyvirus). The broad host range of some phages was partially explained by the presence of receptor-binding protein carrying a polysaccharidase domain. To demonstrate their therapeutic potential, a phage cocktail consisting of eight phages belonging to eight different genera, was tested against BEN4358, an APEC O2 strain. This phage cocktail fully inhibited, in vitro, the growth of BEN4358.

Phages for Global Health (PGH) is training scientists in the region of East Asia to isolate relevant therapeutic phages for pathogenic bacteria within their locality, and thus contributing to making phage technology universally available. During the inaugural PGH workshop in East Africa, samples from Ugandan municipal sewage facilities were collected and two novel *E. coli* lytic phages were isolated and characterized.

The phages, UP19 and UP30 lysed ∼82% and ∼36% of the 11 clinical isolates examined, respectively. The genomes of UP19 (171.402 kb, 282 CDS) and UP30 (49.834 kb, 75 CDS) closely match the genera Dhakavirus and Tunavirus, respectively. The isolated phages have therapeutic potential for further development of treatments against *E. coli* infections [208].

Phages against *Salmonella* Infections

Plasmid-dependent phages infect bacteria carrying conjugative plasmids by recognizing the plasmid-encoded pilus. Two lytic phages from wastewater were isolated using a virulent strain of *Salmonella enterica* carrying the conjugative IncN plasmid pKM101. Both phages, named Lu221 and Hi226, were novel dsDNA viruses within the class Caudoviricetes with genomes of approximately 76 kb. They showed a broad host range infecting *E. coli*, *S. enterica*, *Kluyvera* sp., and *Enterobacter* sp. carrying conjugative plasmids. They recognized plasmid-encoded receptors from 12 out of 15 tested plasmids, all of them carrying resistance determinants [209].

Genomic and phenotypic analysis of *S. enterica* phages identified two novel phage species. The host range, morphology, and genetic diversity of eight *S. enterica* phages isolated from a wastewater treatment plant were assessed. The host range analysis revealed that six out of eight phages lysed more than 81% of the 43 *S. enterica* isolates tested. Whole-genome sequencing (WGS) data revealed that phage genome sizes ranged from 41 to 114 kb, with GC contents between 39.9 and 50.0%. Two of the phages, SB13 and SB28, represent new species, *Epseptimavirus SB13* and *Macdonaldcampvirus*, respectively, as designated by the International Committee for the Taxonomy of Viruses (ICTV) using genome-based taxonomic classification. One phage (SB18) belongs to the *Myoviridae* morphotype while the remaining phages belong to the *Siphoviridae* morphotype. None of the phages possessed virulence, toxin, antibiotic resistance, type I–VI toxin–antitoxin modules, or lysogeny genes (by gene content analyses) [210].

Others

Nakonieczna et al. [211] discovered three novel bacteriophages, J5a, F16Ba, and z1a, specific for *Bacillus anthracis* (potentially highly lethal) by screening environmental samples from various regions in Poland and presented their basic characteristic. The new phages and their closest relative phages, Tavor_SA, Negev_SA, and Carmel_SA, form a separate clade of the *Wbetavirus* genus, were designated as J5a clade. The comparative genomic analysis indicated that the new bacteriophages encode two receptor-binding proteins, of which one may bind a sugar moiety of *B. anthracis* cell surface.

Global warming favored becoming a range of bacteria, such as *Aeromonas hydrophila*, pathogenic to humans. They are not easy for treatment by traditional methods due to their capacity to form biofilms. Bacteriophage offer a possible alternative approach for controlling the growth of the biofilms.

Kabwe et al. [212] first reported the isolation and characterization of bacteriophages which carry intrinsic antibiotic resistance genes and are capable of disrupting biofilms caused by clinical isolates of *A. hydrophila*.

Kallies et al. [213] identified huge phages (with genomes larger than 200 kilobases) from wastewater metagenomes by screening 165 wastewater metagenomes for the presence of viral sequences. The dataset of over 600 identified potential huge phage genomes was reduced using manual curation by excluding those not containing viral protein-coding genes or consisting of concatemers of several small phage genomes. A phylogenomic analysis of the huge phages and phages with smaller genomes (less than 200 kb) supported the hypothesis that huge phages have undergone convergent evolution. The genomes contain typical phage protein-coding genes, sequential gene cassettes for metabolic pathways, and complete inventories of tRNA genes covering all standard and rare amino acids.

##### Bacteriocins

Bacteria produce a range of antimicrobial peptides, the most diverse of which are bacteriocins. Bacteriocins are small antimicrobial peptides (peptide toxins), synthesized by ribosomes of both Gram-positive and Gram-negative bacteria and usually display activity against bacteria (pathogenic and multidrug-resistant), phylogenetically related to the producing strain. The antimicrobial activity spectrum depends on the peptide that can target several bacteria [214,215].

The selectivity and safety profile of the bacteriocins are their superior advantages over traditional antibiotics; however, the bacteriocins are susceptible to degradation by proteolytic enzymes and therefore have low in vivo stability. In addition, their large-scale production is problematic. It is expected that such limitations will be avoided by extensive research, including development novel drug delivery systems [216].

Yount et al. described discovery of Type II bacteriocins, using a new high dimensional bioinformatic algorithm. In this way, all bacteriocin families of Type II were detected whereupon identified putative bacteriocins with broad-spectrum antimicrobial activity against a range of human pathogens. The putative bacteriocin sequences were from different microorganisms: *Bacillus thuringiensis*, *Eubacterium rectale*, *B. cereus* and *Enterococcus pallens* [217].

Bacteriocins from Lactic Acid *Bacteria*

Lactic acid bacteria (LAB) are one of the most used bacteria to produce bacteriocins that could serve as alternatives of conventional antibiotics. *Enterococcus faecalis*, *Lactobacillus fermentum*, *L. plantarum*, *L. helveticus*, *L. pentosus*, *L. paracasei* subsp. *paracasei*, *L. rhamnosus I*, and *L. delbrueckii* subsp. *lactis* are strong strains in bacteriocins production. To date, Nisin, Pediocin PA-1, and Minocin are the only FDA-approved bacteriocins to use as food preservatives [218].

LAB-bacteriocins could be used alone, or as potentiating agents to treat bacterial infections with aim to reduce the use of traditional antibiotics and to develop novel therapeutic options. Most LAB-bacteriocins act by disturbing the cytoplasmic membrane through forming pores, or by cell wall degradation. Some of the bacteriocins that are active against Gram-negative bacteria still have an unknown mode of action. Most bacteriocins-producing strains have an immunity mechanism involving an immunity protein and a dedicated transport system. The immunity mechanisms usually vary from one bacteriocin to another [184].

Lei et al. [219] presented partial purification and characterization of a broad-spectrum bacteriocin, produced and isolated from infant’s feces *Lactobacillus plantarum ZRX03*. The fermentation supernatant, produced by this strain inhibits *E. coli*, *Staphylococcus aureus*, and *Listeria monocytogenes* (inhibition zone of 12.83 ± 0.62 mm, 15.08 ± 0.31 mm, 6.75 ± 0.20 mm, respectively), stronger than the lactic acid bacteria N1, N2, M13, M21, M31, and M37. Ethyl acetate was selected as the optimal crude extract solution. A broad-spectrum antimicrobial activity was shown for the obtained bacteriocin, inhibiting Gram-positive bacteria, Gram-negative bacteria and yeast, including *S. aureus*, *Bacillus subtilis*, *Bacillus anthracis*, *E. coli*, and *Salmonella*, [219]. Circular bacteriocin, plantacyclin B21AG from *Lactiplantibacillus plantarum* B21 (isolated from nem chua, Vietnamese sausage) was discovered with broad-spectrum antimicrobial activity against Gram-positive bacteria, high thermostability and proteolytic resistance [220]. Bacteriocin-like peptide (SLG10), made by *Lactobacillus plantarum* strain, with antimicrobial activity against both Gram-positive and Gram-negative bacteria, was isolated from kombucha (a fermented bubble tea). Stability studies show that the peptide retains its antimicrobial properties for 14 days at 37 °C and for 2 months at 4 °C being stable at pH values between 2.0 and 7.0 [221].

A novel bacteriocin LSX01 of *Lactobacillus paracasei* LS-6 was isolated from a traditional fermented yogurt (produced in Yunnan, China), purified and characterized. The LSX01 exhibits an extensive antimicrobial spectrum against both Gram-positive and Gram-negative bacteria as well as a tolerance to heat, acid-base treatments, and a sensitivity to proteolytic enzymes. The treatment of *S. aureus* planktonic cells with LSX01 significantly reduced their metabolic activity and disrupted the cell membrane integrity. A biofilm formation of *S. aureus* was also significantly inhibited [222].

Thuy et al. [223] characterized the broad-spectrum antibacterial activity of bacteriocin-like inhibitory substance-producing probiotics, isolated from fermented foods. Selected lactic acid bacteria (LAB) with probiotic potential were evaluated by various tests, including exopolysaccharide production, antibiotic susceptibility, acid and bile tolerance, antibacterial activity, and cell adhesion and cytotoxicity to gastric cell lines. Six selected LAB strains demonstrated high viability under gastrointestinal conditions, produced high exopolysaccharides, showed no or less cytotoxicity, and adhered successfully to gastric cells. Three strains, *Weissella confusa*, *Lactiplantibacillus plantarum*, and *Limosilactobacillus fermentum*, demonstrated a strong antibacterial effect against drug-resistant *Escherichia coli*, *Klebsiella pneumoniae*, *Pseudomonas aeruginosa*, *Salmonella enterica serovar Choleraesuis*, *Enterococcus faecium*, and *Staphylococcus aureus*. The whole genome sequencing of these three strains (using the Nanopore platform) showed that they do not harbor genes related to toxins, super antigens, and acquire antimicrobial resistance. The extract of CYLB30 and CYLB47 bacteriocin-like substances (BLIS) inhibited the growth and biofilm formation of drug-resistant *P. aeruginosa* and methicillin-resistant *S. aureus*, causing membrane disruption and inhibiting adhesion ability to human skin HaCaT cells.

*Lacticaseibacillus paracasei*-derived antibacterial peptide NGJ1D was found in the fermentation broth of *L. paracasei*. The antibacterial peptide NGJ1D had minimum inhibitory concentration (MIC) of 62.5 μg/mL against *Staphylococcus aureus* and could kill the bacteria within 3 h [224].

*Enterococcus faecalis* (CAUM157), a Gram-positive bacteria isolated from raw cow’s milk, was studied for its bacteriocin production. In vitro and in silico characterization of N-formulated, di-peptide bacteriocin from *E. faecalis* was lately presented with anti-*Listeria* activity. The antimicrobial activity of CAUM157 was attributed to a two-peptide class IIb bacteriocins with potent activity against food-borne pathogen *Listeria monocytogenes* and periodontal disease-causing pathogens (*Prevotella intermedia* KCTC 15693 ^T^ and *Fusobacterium nucleatum* KCTC 2488 ^T^). Although *E. faecalis* CAUM157 innately has genes for virulence factors and antibiotic resistance (e.g., tetracycline and erythromycin), its bacteriocin production is valuable for the needs of in live microorganisms and pathogens control [225]. Cui et al. [226] perform purification and characterization of novel bacteriocins, produced by *E. faecalis* CG9 from human saliva that inhibit the growth of Gram-negative bacteria. A study of one of the isolated *E. faecium* bacteriocins, Plantacyclin B21AG, showed its excellent stability and bactericidal activity against sporulating bacteria, such as *Clostridium perfringens* and non-sporulating *Listeria monocytogenes*. Sharma et al. [227] found that the bacteriocins produced by *vaginal E. faecium* enterocin 12 A inhibits multidrug-resistant Gram-negative bacteria such as *Salmonella enterica*, *Shigella flexneri*, *E. coli*, and *Vibrio cholerae*, as well as the proliferation of cancer cells.

Bacteriocines from *Staphylococci*

Newstead et al. [228] isolated several bacteriocins from commensal coagulase-negative *Staphylococci*, many of which displayed in vitro and in vivo inhibiting activity against *S. aureus*. The ability of these bacteriocins to destroy a biofilm formation, their novel mechanisms of action and efficiency against antibiotic-resistant bacteria make them novel antibacterial candidate therapeutic. Ovchinnikov et al. [229] reported successful development of bacteriocins into therapeutic formulation for treatment of methicillin-resistant *Staphylococcus aureus* (MRSA) skin infection in a murine model. The potential of two broad-spectrum bacteriocins, garvicin KS and micrococcin P1, were explored in this study for skin infection treatments. The two bacteriocins act synergistically with each other and with penicillin G in killing MRSA in vitro. To assess its therapeutic potential, the three-component formulation was involved in a murine skin infection model with a multidrug-resistant luciferase-tagged MRSA Xen31, a strain derived from the clinical isolate *S. aureus* ATCC 33591. The efficiency of the three-component formulation, in eradicating the pathogen from treated wounds was demonstrated by using of the tagged-luciferase activity as a reporter for the presence of Xen31 in wounds. As compared to Fucidin cream, which is an antibiotic commonly used in skin infection treatments, this formulation appears to be superior in terms of preventing the development of resistance [229].

Bacteriocines from *Streptomyces*

Exploring antibacterial properties of bioactive compounds, isolated from *Streptomyces* sp. in bamboo rhizosphere soil (collected within the Megamalai forest of the Western Ghats in the Theni zone of Tamil Nadu, India) was recently presented. Bioactive compounds were extracted from the culture medium using ethyl acetate. Their antibacterial and antioxidant activities were evaluated through disc diffusion and DPPH radical scavenging methods, respectively. Ethyl acetate extracts were analyzed by FT-IR and GC–MS techniques. Among nine total strains of *Actinobacteria*, the strain BS-16 identified as *Streptomyces* sp., displayed remarkable antibacterial activity against three strains: *S. aureus*, *B. subtilis*, and *Streptococcus pyogenes* with inhibition zone of 19 mm, 12 mm and 10 mm respectively [230].

#### 3.2.4. Approaches to Address Shortcomings of Natural Antibacterial Peptides

Shortcomings, such as low stability, high toxicity in some cases, lability to proteolytic degradation, poor pharmacokinetics, high production costs, as well as difficulties in the specific targets design of successful in vitro natural antibacterial peptides (ABPs), continue to hamper their clinical applicability [145]. Existing experimental and computational tools attempt to address some shortcomings of the ABPs and to ease the preclinical and clinical development of novel therapeutics based on antimicrobial peptides [151]. Use of nano-delivery systems, as well as modification or synthesis of new AMPs inspired by nature are some of them.

Mwangi et al. [231] discussed key strategies used to optimize the performance of ABPs, including rational design and de novo synthesis as well as predictive computational tools and utilizing artificial intelligence in the design and synthesis of highly efficient lead drug candidates. Research on discovery and optimization of lead molecules, originating from natural sources [232] and nanotechnology-based delivery are some of them [145]. Luong et al. [233] discussed approaches to improve biological activity of ABPs either by modifying chemical structure or incorporating into delivery systems.

##### Nano-Delivery Systems

Nano-delivery is known to enhance the antibacterial efficacy of classical antibiotics and ABPs, while reducing their toxicity due to its capability to improve the bioavailability and permeation across barriers of classical antibiotics and ABPs, and/or to protect against degradation. The biological activity of nano-delivery systems could be time-saving and cost-effective predicted by machine learning [234,235].

The cyclic peptide antibiotic, polymyxin B (produced by *Bacillus polymyxa* bacterium) is an example of improved biological activity by nano-delivery. Polymixin B cross-linked with sodium alginate in polyion complex nanoparticles or loaded in niosomes show enhanced bioavailability and increased potential antimicrobial activity against *Pseudomonas aeruginosa* [236].

Stan et al. [23] reviewed nano-carriers used for transportation of natural antibacterial compounds to improve their stability, bioavailability, cellular uptake/internalization, pharmacokinetic profile and to reduce the toxicity of these natural compounds. There are a number of nanocarriers such as liposomes, micro-emulsion systems, nanocapsules, solid lipid nanoparticles, polymeric micelles, and dendrimers. However, some recent studies are focused on the incorporation of natural antimicrobial substances into polymeric nanoparticles, niosomes and silver nanoparticles, having intrinsic antimicrobial activity, are some of them. Many recent in silico or in vitro works show that the internalization of optimal nanocarriers represents the future of the “green therapeutics”.

ABPs are susceptible to proteolytic degradation in body fluids and therefore the tablet, capsule, and solution are inconvenient when given orally due to peptide degradation and lower absorption in the gastrointestinal tract. Special drug delivery systems and formulations like nanocarriers, hydrogels, cubosomes, wafers, dry powder inhalers, creams, and mouth rinses were established to prevent some major barriers to clinical applications, such as cytotoxicity, bioavailability, and stability. Several specialized drug delivery systems of AMPs were already approved by FDA and some others are under clinical trials [137].

##### Modified and Synthetic Analogues of Antibacterial Peptides Inspired by Nature

It was experimentally found that natural peptides with special chemical structure demonstrate high antibacterial activity, and some modifications of the peptide backbone and structure can additionally improve their biological activity and stability as well as reduce the toxicity of these ABPs. Inspired by nature, development of modified and synthetic analogues of natural ABPs is an extensive current approach that is based on different chemical strategies assisted by in silico research. Gan et al. [237] presented various chemical synthetic strategies to develop ABPs with improved properties, including chemical modifications of existing peptides, semi-synthesis, and computer-aided design. Novel ABP structures, including hybrid, dendrimer and polypeptide, peptidomimetic, and ABP–drug conjugates were also highlighted. Zhou et al. [238] demonstrated that poly(2-oxazoline) (POX) can work as a functional mimic of peptides. POX-based glycine pseudo-peptides, a host defense peptide mimic, have potent activities against methicillin-resistant *S. aureus*, which causes formidable infections. *S. aureus* does not develop resistance to POX owning to the reactive oxygen species related antimicrobial mechanism.

It is thought that understanding the AMPs structure–function relationship has potential to help the conceptualization of the development of new ABPs by evaluation the role of each residue and essential amino acids for their biological activity. This feature could help the development of second-generation AMPs with high potential antimicrobial and other activity [239]. Li et al. [6] summarized recent synthetic efforts, their impact on analogue design and various applications in the development of ABPs for next generation antimicrobial agents. Focusing on antimicrobial peptidomimetics, they presented modifications to enhance antimicrobial activity, such as lipidation, glycosylation and multimerization through a broad application of novel bio-orthogonal chemistry.

Ajish et al. [240] designed novel short antimicrobial peptide BP100-W with antimicrobial, anti-biofilm and anti-inflammatory activities by replacement with tryptophan. Wiman et al. [241] developed novel broad-spectrum antimicrobial lipopeptides derived from plantaricin NC8 β. Chaudhary et al. [242] reported efficient production of bioactive amidated ABPs by transient expression of glycine-extended ABPs in *Nicotiana benthamiana* line expressing the mammalian enzyme peptidylglycine α-amidating mono-oxygenase (PAM). Cationic ABPs accumulate to substantial levels in PAM transgenic plants compared to non-transgenic *N. benthamiana*. Moreover, ABPs purified from plants exhibited robust killing activity against six highly virulent and antibiotic-resistant ESKAPE pathogens, preventing their biofilm formation, analogous to their synthetic counterparts and synergized with antibiotics. The experimental data and techno-economic analysis demonstrated the potential use of plant chassis for large-scale production of clinical-grade ABPs [243]. The study of antimicrobial cyclic peptides has become a hot topic. Among them, macrocyclic AMPs have received extensive attention. The structures and functions of the dominant cyclic natural and synthetic AMPs were presented with an outlook on the future direction of cyclic AMPs in [144].

Many databases were created to collect both fundamental and pharmacological information summarizing sources, structures, modes of action, and classifications of ABPs, valuable computational tools for prediction the antimicrobial activity and new machine learning approaches that can be employed to improve ABP activity. Multi-label classifiers with binary relevance and algorithm adaptation techniques are used to predict different functions of ABPs across a wide range of pathogen bacteria. Forward feature selection identifies sequences order and charge as critical, with specific amino acids as discriminative. These findings provide valuable insights for the design of ABPs with multiple functionalities, thus contributing to the broader effort to combat drug-resistant pathogens [139]. Peptide databases and different computational tools are available as resources to collect ABPs and beneficial tools for prediction and design of computational models for new biologically active AMPs [243].

The computer-aided design puts together crucial information on chemical parameters and bioactivities in AMP sequences, thus providing modes of prediction to evaluate the antibacterial potential of a candidate sequence before synthesis. Quantitative structure-activity relationship (QSAR) computational models, for instance, greatly contribute to AMP sequence optimization aimed at improved biological activities. In addition to machine-learning methods, the de novo design, linguistic model, pattern insertion methods, and genetic algorithms, show a potential to boost the automated design of AMPs. As expected from automated design strategies, diverse candidate sequences with different structural arrangements were generated and deposited in the databases [244].

Boone et al. [245] presented combining genetic algorithm with machine learning strategies for designing potent antimicrobial peptides with an example of supervised machine learning and a genetic algorithm to find a peptide active against *S. epidermidis* (a common bacterial strain for implant infections) with an improved aggregation propensity average for an improved ease of synthesis. This was the first time that codon-based genetic algorithms combined with rough set theory methods was used for computational search on peptide sequences.

C. Rumancev et al. [246] demonstrated that the emerging BioSAXS method could help to elucidate the mode of action of ABPs that contribute to the development of antibiotics against resistant bacteria. The effect of two highly active short, broad-spectrum ABPs (14D and 69D) was investigated against *E. coli* and methicillin-resistant *S. aureus* (*MRSA*), as well as further studied the ultrastructural changes in *E. coli* and MRSA in response to these AMPs using the BioSAXS technique.

Jianxun et al. prepared α-Helical antimicrobial peptide YHX-1 by de novo design and estimated its antibacterial activity. To obtain a novel antimicrobial peptide, the sequence length, charge numbers, and amino acid composition of this antimicrobial peptide were determined and optimized based on the existing database of antimicrobial peptides. The newly designed antibacterial peptide YHX-1 is broad-spectrum bactericidal efficient and safe [247].

The results of peptide-protein docking study of Mustafa et al. [248], based on 10 selected ABPs, among which napin and snaking-1, confirmed by molecular dynamics simulations, demonstrates that the peptide-protein docking computational approach supports the evidence of efficiency of these ABPs as potential inhibitors of bacterial strain specific proteins. Lin et al. [249] performed intelligent de novo design of novel antimicrobial peptides against antibiotic-resistant bacterial strains. They trained a Wasserstein generative adversarial network with gradient penalty (WGAN-GP) based on known ABPs to generate novel ABP candidates. The quality of the GAN-designed peptides was evaluated in silico, and eight of them were selected by an ABP Artificial Intelligence (AI) classifier and synthesized for further experiments. This approach showed an efficient way to discover ABPs effective against general and antibiotic-resistant bacteria strains. Such a strategy allowed other novel functional peptides to be quickly designed, identified, and synthesized for validation on the wet bench [249].

Szymczak et al. [250] proposed a novel approach to a potent antibacterial peptide generation, using a deep generative model HydrAMP (that is a cVAE-based model), which was specifically trained to perform analogue generation of both positives and negatives, as well as unconstrained generation. Nedyalkova et al. [251] presented the progress and future of the computational design of antimicrobial peptides (ABPs) as bio-inspired functional molecules. The latest scientific breakthroughs and technologies that could offer new opportunities and alternative strategies for developing novel ABPs are in the focus of this discussion.

Aguilera-Puga et al. [252] discussed the evolution and applications of predictive and generative modelling to discover and design safe and effective antimicrobial peptides as well as accelerating the discovery and design of antimicrobial peptides with artificial intelligence. Peptides, which modulate many processes of human physiology (targeting ion channels, protein receptors, or enzymes) represent valuable starting points for the development of new biologics against communicable and non-communicable disorders. However, turning native peptide ligands into drug gable materials requires high selectivity and efficacy, predictable metabolism, and good safety profiles.

### 3.3. Biogenic Synthesized Metal, Metal Oxide and Composite Nanoparticles

The use of nanomaterials is accepted as promising approach in the fighting of bacterial infections and antibiotic resistance. Unlike the antibiotics, which are administered to treat diseases and infections in the patients, the nanomaterials provide an opportunity to limit microbial growth prior to human infection [253]_._

Many novel antibacterial nanomaterials were developed during recent years and some of them are already applied in some hospitals [254]. The experimentally proved remarkable antimicrobial activity of metal and metal oxide nanoparticles as well as of metal nanocomposites seems to be very interesting for alternative treatment of multidrug-resistant microbial infections [255,256]. The nanoparticles (NPs) are small enough to pass through the cell membranes of pathogenic bacteria and interfere with essential molecular pathways [256]. The antibacterial activity of the metallic nanoparticles (NPs) is accepted to be due to interactions with important cellular organelles and biomolecules like DNA, enzymes, ribosomes, and lysosomes that can affect cell membrane permeability, oxidative stress, gene expression, protein, and enzyme activation [257]. Since the NPs target multiple biomolecules concurrently, it becomes difficult for the bacteria to develop resistance against them [258].

NPs could be utilized by their including in delivery systems, as intrinsic antimicrobials, or in mixtures. The techniques for obtaining are very important for the properties of the produced metal NPs and hybrid nanostructured materials, the particle size and distribution on size being among the most important ones [259].

Until now, the developed synthesis methods are of three categories: physical, chemical, and biological. Physical methods use a top-down strategy, i.e., a large piece of metal is fragmented into small parts by physical action into progressively smaller fragments. NPs are produced with a somewhat scattering size distribution. The NPs size mainly determines their antibacterial activity. Therefore, the physical methods are not preferable for preparation of metallic NPs. Chemical methods are based on bottom-up strategies leading to formation of more narrow size distributed metallic NPs. They utilize chemical procedures that use chemical solvents and certain drawbacks such as toxicity and others limit their utilization in the synthesis of metallic NPs. The biosynthesis does not use toxic chemicals, it is eco-friendly and therefore it is favored among the other methods of NPs creation [256].

The biogenic synthesis of metal NPs receives global attention as an eco-friendly and cost-effective approach. Some microbes were shown to naturally produce metal-based NPs as a method for the detoxifying of heavy metals [260]. However, the biogenic synthesis of metal NPs has been investigated in detail in recent decades, and since then, metal-based NPs find application in the production of cosmetics and textiles. The adaptability of these substances attracts the world’s scientific attention, and the development of novel formulations, applications, and synthesis techniques continue [256].

It is known that living organisms like bacteria, yeast, fungi, and plants can reduce inorganic metal ions into metal NPs by their cellular metabolites and thus to produce various metal and metal oxide NPs. This is the base of the current biogenic synthesis of NPs. Both the yield and stability of the biogenic NPs are quite satisfactory. Photosynthesis of NPs from plants seems to be a very effective way for the development of a rapid, clean, nontoxic, and eco-friendly technologies for obtaining of a variety of biologically active nanomaterials for medical applications [258].

Extracts from different plant species are used for biosynthesis of NPs, such as silver (Ag), cerium dioxide (Ce_2_O_2_), copper oxide (CuO), gold (Au), titanium dioxide (TiO_2_), and zinc oxide (ZnO). Plant extracts could reduce the use of hazardous compounds and harsh reactions in the production of metallic NPs [261]. Plant, fungal, algal, and cyanobacterial extract solutions were employed as nucleation/capping agents in the green bioprocess to produce effective nanomaterials for medical applications. Biogenic synthesized NPs show wound-healing antibacterial, anticancer, and antifungal properties [262]. The biogenic syntheses of metallic NPs from seed extracts are presented as a cost-effective alternate to other biological methods due to low maintenance costs, culture/growth independence for biomass, and diversity of phytochemicals as reducing and capping agents. Recently, seed extracts have become popular because of the single-step green synthesis of metal NPs with satisfactory antimicrobial activity [263].

The utilization of phytochemicals from plant extracts has become a unique technology for the synthesis of NPs, as they possess dual nature of reducing and capping agents to the NPs. Green synthetized NPs attract extensive interest worldwide because of their biocompatibility and huge potential for utilization as antimicrobial agents, as well as in cancer/gene therapy, and in energy harvesting. The biological reduction of metals provides NPs of desirable size and morphology with better physicochemical characteristics, lower toxicity, and high stability. Fungi are the most preferred for the biogenic synthesis of NPs due to high biomass production, secretion of a high quantity of extracellular proteins that stabilize the NPs, ease in handling, and high metal tolerance [264,265].

Currently, NPs synthesized using plant or microbial extracts and conjugated with biologically active components are discussed as a safe alternative approach for therapeutic applications as they are environmentally friendly and cost-effective. Furthermore, NPs conjugated with natural biomolecules have improved bioavailability and minimal side effects, as they are smaller in size and have higher permeability in addition to being reducing and stabilizing agents possessing excellent antioxidant activity. NPs serve as potential antimicrobial agents due to their affinity towards sulfur-rich amino acids, adhere to microbial cell walls by means of electrostatic attraction, and disrupt the cytoplasmic membrane along with the nucleic acid of microbes [266].

Bhati [267] described the green methods for the synthesis of biogenic NPs, involving various plant extracts which are nontoxic, environmentally friendly and value effective. Natural plant extracts contain metabolites like flavonoids, terpenoids, polyphenols, and alkaloids which act as both reduction and stabilization agents for synthesis of biogenic NPs with desired shape and size. Furthermore, the employment of assorted plant extracts and bacteria, fungi, and proteins within the biogenic synthesis of NPs are described shortly. It is anticipated that transition metals will be the ideal choice for a production of metal-based nanoparticles because they have partially filled d-orbitals, which makes them more redox-active [268].

Numerous approaches to green synthesis of different metal NPs, metal oxide NPs, and nanocomposites by using a variety of natural reducing agents originating from terrestrial biota are described in the special literature. Strategies for improving the antibacterial capability of the NPs through surface modification and their potential for applications in antimicrobial performance targeted at eradicating multidrug-resistant bacteria are also investigated. Although research was recently extended to less-common metals, the materials most extensively studied and used in metal-based NPs, remain silver, gold, copper, iron, and zinc.

#### 3.3.1. Biosynthesized Silver Nanoparticles

Due to their intrinsic therapeutic properties and the broad-spectrum antibacterial activity, the silver NPs were recognized as potential candidates for development of novel approaches to the treatment of various bacterial diseases [269,270,271]. Their bactericidal activity is associated with a triggering the oxidative stress, protein dysfunction, membrane and DNA damage that leads to damage of the microbial cells [272]. The size, shape, and concentration play an important role in the antimicrobial activity of the silver NPs. As antibacterial agents, the silver ions are usually more effective than the silver NPs. However, modified or functionalized silver NPs could be extremely active to kill bacteria than pure silver NPs [273]. For example, the modification of silver nanoparticles with titanate nanotubes changes their physicochemical properties (such as stability, size, oxidation state, and shape), resulting in enhanced antibacterial, catalytic, and photocatalytic activity [254]. Exhibiting multiple and simultaneous mechanisms of action, the silver NPs are active against Gram-negative and Gram-positive bacteria, including multidrug-resistant strains. In combination with antibiotics or organic compounds as antibacterial agents, they demonstrate synergistic effect against pathogen bacteria such as *Escherichia coli* and *Staphylococcus aureus* [274].

Different approaches to green synthesis of silver NPs by use of natural reducing agents originating from the terrestrial biota were presented in the literature. Gupta et al. [275] accomplished green synthesis of silver NPs for wound dressing applications using curcumin-cyclodextrin complexes, loaded into bacterial cellulose-based hydrogels. Curcumin, that is a natural polyphenolic compound (well known as a wound-healing agent) is used as a natural reducing agent. The hydrophobicity of the curcumin overcomes by its microencapsulation in cyclodextrin. These novel dressings exhibit antimicrobial activity against three common wound-infecting pathogenic microbes: *S. aureus*, *Pseudomonas aeruginosa*, and *Candida auras*. Das et al. [276] highlighted silver NPs synthesis mediated by plant and algae derivatives and discussed their application as antibacterial agents with emphasis of their role for providing further better health, environment and prevention from infectious diseases. Feroze et al. [277] performed fungal mediated biosynthesis of silver NPs in the presence of fungal metabolites of *Penicillium oxalicum* (characterized by XRD and SEM). The evaluation of the antimicrobial activity (by disc diffusion test and UV-Visible Spectrophotometry) of the biosynthesized silver NPs, demonstrates their excellent activity against *Staphylococcus aureus*, *S. dysenteriae*, and *Salmonella typhi* and indicates that such silver NPs might be useful as bactericidal agents against drug resistant bacteria. Garibo et al. [278] found that green synthesized silver NPs by using *Lysiloma acapulcensis* (a perennial tree used in the traditional Mexico medicine) exhibit higher antimicrobial activity than that of chemically produced silver NPs, maintaining their low-cytotoxicity. The obtained antimicrobial potency was as follows: *E. coli* ≥ *S. aureus* ≥ *P. aeruginosa* > *C. albicans*. FTIR and LC–MS results show the presence of chemical groups that could act as either (i) reducing agents stabilizing the silver NPs, or (ii) antimicrobial capping agents enhancing antimicrobial properties of silver NPs. Rahimi et al. [279] presented recent progress in the antimicrobial wound dressings based on carbohydrate polymer-based silver nanocomposites. The methods of synthesis, physicochemical properties, healing efficiencies, toxicity against human tissues, antibacterial and antifungal effects of each material were discussed. Barabadi et al. [280] used simple, non-toxic and fast method to fabricate *Zataria multiflora*-derived silver NPs. The antibacterial activity evaluation against *Staphylococcus aureus* demonstrated the higher activity of the plant-mediated fabricated silver NPs as compared to that of commercial silver NPs. Ramires-Rosas et al. [281] presented a green route to produce silver NPs using the bioactive flavonoid quercetin as a reducing agent and food anti-caking agents as stabilizers.

Chavan et al. [282] described a green synthetic approach to produce silver NPs using an *Artocarpus heterophyllus* leaf extract and Design Expert Ver. 13 to optimize their parameters. The optimized silver NPs were characterized by UV–Vis and FTIR spectroscopy. Antioxidant and antimicrobial potential were determined in vitro using standard protocols. The optimized nanoparticles appeared to be spherical, with average particle diameter of 100–110 nm (SEM, TEM) and showed effective antibacterial, antioxidant, and antifungal activity.

Mahalingam et al. [283] synthetized biogenic silver NPs with a spherical shape, average particle size of 15 to 25 nm, that were stable and monodispersed, using ethanol extract of *Catharanthus roseus* flower. UV–Vis and FTIR spectroscopy, XRD, Particle Size Analysis, TEM, and EDX characterized the NPs. These silver NPs revealed superior antibacterial activity against human pathogenic bacteria with a remarkable inhibition zone for *Salmonella typhimurium* (10–14 mm), *Bacillus* subtilis (6–11 mm), for *Enterococcus faecalis* (11–14 mm) and *Shigella boydii* (9–10 mm).

Said et al. [284] synthetized silver NPs which were active against the common pathogens for urinary tract infections, using Egyptian henna leaves (*Lawsonia inermis*) extracts. Plant extract components were identified by GC-MS and the analysis of the prepared silver NPs was carried out through UV–Vis, XRD, TEM, SEM, and FTIR spectroscopy. Antibacterial activities of the obtained silver NPs were examined against common pathogens from urinary tract. Very high sensitivity of all test pathogens to the biologically synthesized silver NPs was observed.

Hani et al. [285] used orange peel extract in a biogenic synthesis of silver NPs with aim to utilize an agro-industrial byproduct, specifically *Citrus sinensis* peels, as a reservoir of polyphenols. The natural chemicals presented in *C. sinensis* peels served to reduce agents in an environmentally benign method for synthesis of silver NPs. The latter were characterized by UV–Vis spectroscopy, Dynamic Light Scattering (DLS), SEM, EDX, and TEM. Their effectiveness in inhibiting growth and biofilm formation of *E. coli*, *Staphylococcus aureus*, and *Candida albicans* was demonstrated simultaneously with significant toxic effects against human prostate cancer cell line DU145 (as investigated by anti-apoptotic, 4′,6-diamidino-2-phenylindole (DAPI), reactive oxygen species (ROS), and acridine orange/ethidium bromide (AO/EtBr) assays). All findings confirm that this approach can serve as a cost-effective, non-toxic, and environmentally friendly technology for green synthesis of medical silver NPs, that offers an alternative recycling strategy and contributes to the sustainable use of biological by-products.

Silver/silica nanocomposite (Ag/SiO_2_) was biosynthesized at room temperature using the crude extract of *Escherichia coli* metabolites in presence of sunlight. It was characterized by UV-Vis spectrophotometry, XRD, FTIR and TEM. The Ag/SiO_2_ NPs show average size of ~32–48 nm whereas silver NPs show a mean size of 18–24 nm. The negative charged Ag/SiO_2_ (−31.0 mV) indicate potential antimicrobial activity against *Bacillus cereus*, *Klebsiella pneumoniae*, *Staphylococcus aureus*, *E. coli*, *Candida albicans*, and *Botrytis cinerea*. The minimum inhibitory concentration (MIC) test shows a dose-dependent antimicrobial action of the Ag/SiO_2_ NPs. In addition, a formation of a mucilage matrix connecting the hyphal cells were observed as well as a big vacuoles and lipid droplets appearance with severe leakage of cytoplasmic contents of the treated *B. cinerea* [286].

#### 3.3.2. Biosynthesized Zinc Oxide Nanoparticles

Naseer et al. [287] developed a green route to synthesize zinc oxide nanoparticles (NPs) using leaf extracts of *Cassia fistula* and *Melia azadarach* and prove their antibacterial potential. Gur et al. [288] also presented green synthesis of biogenic zinc oxide NPs, their characterization and bioactivity evaluation. Since ZnO and TiO_2_ have no impact on human cells, NPs-based wound care solutions by utilizing these two oxides are presented as relatively new approach compared to conventional materials. Veselova et al. demonstrated the long-term antibacterial efficacy of textiles coated with ZnO and TiO_2_ nanoparticles in a tropical environment [289]. Biogenic synthesis and characterization of ZnO nanoparticles for degradation of synthetic dyes were performed as a sustainable environmentally clean approach [290].

Green synthesized ZnO and vanadium-doped ZnO nanoparticles using *Vinca rosea* plant leaf characterized by FTIR, XRD, and SEM-EDX. Their testing for biomedical applications (antibacterial, anticancer, antioxidant and antidiabetic) showed the higher antioxidant, antidiabetic, and anticancer activity of *Vinca rosea* capped ZnO NPs than vanadium-doped ZnO NPs, both prepared by one the same green approach [291].

ZnO NPs and *Sesbania grandiflora* are known for their biocompatibility and medicinal properties, such as anti-cancer, anti-microbial, anti-diabetic, and anti-oxidant. Ramasubbu et al. [292] presented green synthesis of ZnO NPs using *S. grandiflora* and the evaluation of antimicrobial, antidiabetic and cytotoxic effects.

#### 3.3.3. Biosynthesized Coper Nanoparticles

A study on the biosynthesis of copper nanoparticles (NPs) mediated by a rare medicinal plant *Cissus arnottiana* was reported together with the antibacterial activity against Gram-negative and Gram-positive bacteria. The biosynthesized coper NPs show high antibacterial activity against the Gram-negative bacterium, *E. coli* with an inhibition zone of 22.20 ± 0.16 mm at 75 μg/mL [293].

A strategy for the synthesis of coper NPs was developed that is based on the use of enzymes as stabilizing agents generating metal-enzyme nanobiohybrids. The enzymes facilitate the in situ formation of NPs under mild synthesis conditions (water medium and room temperature) in absence of reducing agents. Furthermore, the use of a protein allows a control over the size and shape as well as over the production of monodisperse metal NPs [294].

Various physical, chemical, and biological methods used for CuO NPs preparation as well as affected by them physicochemical and biological properties of the synthetized CuO NPs are under discussion regarding potential biomedical applications [295]. A recent study of Ortega-Nieto et al. [296] demonstrated the effect of a reduction step inclusion in the coper nano biohybrids synthesis. Changes in the metal structure and antibacterial efficiencies were observed that depend on the added amount of reducing agent. Copper sulphate as a metal salt, lipase from *Candida antarctica* (CAL-B) as a scaffold enzyme, and sodium borohydride (NaBH_4_) as a reducing agent were used. Hybrids were synthetized by using of different percentages NaBH_4_ from 0% to 100%. XRD and TEM analyses demonstrate different oxidation states of the copper and sizes for the coper NPs depending on the degree of reduction. Predominant Cu(0), larger NPs (with a maximum size of 13 nm) and agglomerated nanohybrids were found at higher NaBH_4_ amounts whereas at lower NaBH_4_ amounts, Cu(I) species and smaller NPs (particle size less than 6 nm) are predominant.

#### 3.3.4. Biosynthesized Iron Oxide Nanoparticles

Biosynthesis of iron oxide NPs via a composite of *Psidium guavaja–Moringa oleifera* is reported as a benign, facile, biocompatible, cost-effective and eco-friendly approach. The antibacterial and photocatalytic studies (FTIR, UV-Vis, XRD, TEM and VSM, Vibrating Sample Magnetometer) demonstrate that the in this way synthetized FeO NPs inhibit the growth of six human pathogens with higher activity at lower concentrations compared to the orthodontic antibacterial drugs [297].

#### 3.3.5. Biosynthesized Selenium Nanoparticles

Biogenic selenium NPs were synthesized using herbal extract of *Triphala*. The assessment of their antimicrobial and antioxidant activity and toxicity indicates that the *Triphala*-augmented biogenic selenium NPs have a great potential as oral antimicrobial agents with superior biocompatibility and antioxidant properties. The antimicrobial activity was assessed against *Streptococcus mutans*, *S. aureus*, *E. faecalis* and *Candida albicans* by agar diffusion test, and DPPA assay was used to evaluate the antioxidant potential of the selenium NPs. Their toxicity was tested on brine shrimp [298].

#### 3.3.6. Others

Combinations of metal NPs and other antibacterial agents, like farnesol for example, are also studied as a potential tool to mitigate the problem of AMR. The assessment of the effectiveness of antibacterial agents, composed of farnesol and nanoparticles (silver, gold, copper, and zinc oxide), in the degradation of biofilms, produced by pathogenic microorganisms (*Escherichia coli*, *Enterococcus faecalis*, *Staphylococcus aureus*, *Pseudomonas aeruginosa*, and *Candida albicans*) demonstrates the great changes in the biofilm structure caused by Ag-farnesol composite altering the process of biofilm formation [299].

Biogenic synthetized metal and metal oxide NPs with their narrow particles size distribution, large surface area-to-volume ratio and high biological activity offer numerous options for development of antibacterial agents to treat bacterial infections [300].

## 4. Concluding Remarks

Terrestrial biota derived from antibacterial products and substances open a variety of new ways to improve modern therapeutic strategies. Natural products serve as key sources of novel chemical diversity as well as integral components of currently accessible drug formulations. Originating from plants, animals, and microbes, they have a wide range of chemical and functional variability and show great efficiency for treating infectious diseases with fewer side effects, synergy, and capacity to combating drug tolerance.

New terrestrial sources of known antibacterial agents and new antibacterial agents from terrestrial biota were discovered during the last 5 years, which are under investigation together with long known but now undergoing a renaissance for the development of new medical treatments. Some natural terrestrial biota derived from antibacterial materials and based on antibacterial agents are more efficient, and with lower side effects than their chemically synthesized counter parts.

Development of novel derivatives that avoid some disadvantages of the natural antibacterial compounds and modification of natural products to adjust their properties to different medical applications using natural led molecules and mashing design were performed for the creation of new pharmaceutical products active against different bacterial pathogens including against multidrug-resistant bacteria.

Well-known herb and medicinal plant extracts as well as bacteriophages are now experiencing a renaissance as a base for the development of antibacterial drugs, antibiotic adjuvants, and individual therapies. In the area of plant drug discovery, much progress has been made in the generation and analysis of large chemical datasets.

Special efforts were devoted to the search for novel antibacterial peptides and bacteriophages as well as for biogenic synthetized metal, metal oxide, and composite nanoparticles. A number of new antimicrobial peptides and their modified variations, with in vitro proved high antibacterial activity are adjusted/in adjustment for development of drugs for specific bacterial diseases, some of them being in clinical trials. The peptide antibiotics are already accepted as one of the most important tools to combat multidrug-resistant bacteria. Bacteriophages, especially designed for concerned individuals, are another hope for successful treatment of heavy multidrug-resistant infections.

Antibacterial activity of biogenic synthetized metal, metal oxide, and composite nanoparticles is very promising and some of them are already in clinical application, for example in healing of wounds, orthopedic coatings, among others.

The use of engineered nanomaterials, such as drug delivery systems containing natural antibacterial substances leads to increased efficiency of the antibacterial treatments improving the availability, pharmacokinetic and others.

## Data Availability

This is a review paper, and no new data were created.
